# Designed Polyurethanes for Potential Biomedical and Pharmaceutical Applications: Novel Synthetic Strategy for Preparing Sucrose Containing Biocompatible and Biodegradable Polyurethane Networks

**DOI:** 10.3390/polym11050825

**Published:** 2019-05-07

**Authors:** Lajos Nagy, Miklós Nagy, Bence Vadkerti, Lajos Daróczi, György Deák, Miklós Zsuga, Sándor Kéki

**Affiliations:** 1 Department of Applied Chemistry, Faculty of Science and Technology, University of Debrecen, Egyetem tér 1, H-4032 Debrecen, Hungary; nagy.lajos@science.unideb.hu (L.N.); miklos.nagy@science.unideb.hu (M.N.); bencevadkerti94@gmail.com (B.V.); deak.gyorgy@science.unideb.hu (G.D.); zsuga.miklos@science.unideb.hu (M.Z.); 2 Department of Solid State Physics, Faculty of Science and Technology, University of Debrecen, Bem tér 18/b, H-4026 Debrecen, Hungary; daroczi.lajos@science.unideb.hu

**Keywords:** poly(ε-caprolactone), 1,6-hexamethylene-diisocyanate, sucrose, polyurethane, swelling, mechanical testing

## Abstract

In this paper the preparation and detailed characterization of designed polyurethanes (SPURs) are reported for potential biological, biomedical and/or pharmaceutical applications. Importantly, in order to fulfill these goals all reactants and solvents used were selected according to the proposal of EUR-8 Pharmacopoeia. For the synthesis, a novel strategy was introduced and elaborated. A series of SPUR samples was prepared from poly(ε-caprolactone)-diol, 1,6-hexamethylene diisocyanate and sucrose as a chain extender/crosslinking agent to obtain sucrose containing polyurethanes. In addition, the mol ratios of the sucrose were varied within an order of magnitude. The prepolymers and the products of the syntheses were investigated by matrix-assisted laser desorption/ionization time-of-flight mass spectrometry (MALDI-TOF MS) and infrared spectroscopy (IR), respectively. It was found that the reactivity of the eight free hydroxyl groups of sucrose are different, and after curing the SPUR samples at 60 °C no free isocyanate groups can be observed. Furthermore, swelling experiments performed with various solvents of different polarities revealed that the highest degree of swelling took place in dimethyl-sulfoxide. However, low degrees of swelling were recognized in water and hexane. It is important to note that the gel contents were around 90% in all cases, which demonstrate that the crosslinking was almost complete. In addition, the kinetics of swelling were also evaluated and successfully modeled. The crosslink densities were calculated from the data of the swelling experiments by means of the Flory-Rehner equation. Unexpectedly, it was found that the crosslink density decreased with the increasing sucrose content also in line with the results obtained by relaxation modulus experiments and dynamic mechanical analysis (DMA). The Tg and Tm of SPUR samples, determined from DSC and DMA measurements, were around −57 °C and 27 °C, respectively. According to the mechanical tests the SPUR samples showed high elongation at break values, i.e., high flexibilities. Furthermore, the stress-strain curves were also modeled and discussed.

## 1. Introduction

The last few decades have witnessed an unprecedented increase in human life expectancy and life quality. The key to keep up with these improvements can be offered by tissue engineering and regeneration, which involve the use of a tissue scaffold for the formation of new viable tissue for a medical purpose [1]. The certain mechanical and structural properties the tissues require for proper functioning are to be provided by biocompatible synthetic polymers, such as poly(ε-caprolactone), polyurethanes and polylactic acid, since through macromolecular engineering their properties can be tailored and fine-tuned more precisely than those of natural polymers [1].

Poly(ε-caprolactones) (PCLs) are useful biomaterials whose utilities span from as materials for biomedical devices to food packaging applications. Importantly, the Food and Drug Administration (FDA) has approved PCLs for specific human applications. For example, FDA allowed their use in long term implantable devices, absorbable sutures and in certain drug delivery systems. However, there are some shortcomings with PCLs, such as their low degradation rates and poor mechanical properties, which are not beneficial for their special applications, e.g., for tissue engineering [2,3,4]. One potential solution for improving the physical, chemical and biological properties of PCLs, among others, is to prepare polyurethane derivatives involving PCL blocks.

Polyurethanes (PURs) play an important and unavoidable role in everyday life [5]. They are frequently used as flexible foams, rigid foams, elastomers and so on [5]. The physical, chemical and biological properties of PURs are fundamentally determined by their chemical structures. A general synthetic approach for producing of PURs involves polyaddition reaction between isocyanates and polyols. If the target PURs are intended to be thermosets, the use of crosslinking agent is necessary [5]. The step-growth polyaddition reaction carries a great variability in the compositions including the types and functionality of both the isocyanates and polyols as well as of other additives [5,6,7,8,9]. Furthermore, both isocyanates and polyols can originate from natural resources, such as polyols, e.g., from carbohydrates [6]. Thus, one can vary the chemical structure of PURs in a wide range to tailor their physical, chemical and biological properties for specific applications. The use of naturally occurring carbohydrates for the synthesis of PURs is well-documented [6]. Garcon et al. synthesized PURs from protected sugar derivatives and 1,6-hexamethylene diisocyanate (HDI) [7]. M. Barikani et al reacted corn starch with a prepolymer obtained from the reaction of HDI and poly(ε-caprolactone)-diol (PCLD) to prepare hydrophobic PUR-copolymers [8]. Kizuka and Inonue reported the preparation of PURs from aromatic 4,4’-diphenylmethane diisocyanate (MDI), and different polyols and sucrose as a cross linker were reported by putting all reactants in one pot to obtain copolymers [9]. Since our primary purpose was to synthesize flexible, biocompatible and biodegradable PUR-s for potential biological use in general, and pharmaceutical and/or biomedical applications in particular, we encountered some serious limitations. Importantly, we should exclude all aromatic isocyanates and various unsupported solvents from the synthesis. Thus, the solvents should be selected according to the European Pharmacopoeia (Ph. Eur. 8th edition) requirements, i.e., the solvents should be of class 2 and class 3 [10]. Under these constraints, we elaborated a novel synthetic method to prepare PURs with well-defined structures and networks as well as to control their swellability and flexibility. Our novel concept is based on the followings: (i) first, a prepolymer is synthesized in melt from a biodegradable, biocompatible polyester polyol, i.e., from poly(ε-caprolactone) diol (PCLD) to obtain prepolymer diisocyanate; (ii) in the second step a multifunctional chain extender and/or crosslinking agent dissolved in DMSO is added to the melted prepolymer to obtain the main polymer chain. It is important to note that the substituents of the chain extender/crosslinking agent should have different reactivity and furthermore, the chain extender/crosslinking agent should have two highly reactive functional groups to yield the main polymer backbone chain. Then the reaction proceeds between the excess of free isocyanate groups of the prepolymer and the lesser reactive functional groups in the chain extender/crosslinking agent to form the final polymer network. On the other hand, upon variation of the M_n_ of the polyol moiety in the prepolymer one can vary the distances between the main polymer chains. The crosslink density can thus be regulated deliberately simply by adjusting the molar ratio of the prepolymer and the chain extender/crosslinking agent.

We have recently reported that the reactivity of the primary OH groups towards isocyanates is higher than that of the secondary ones [11]. As it is well known, the sucrose has eight free OH-groups, from which 3 of them are primary OH groups (one of them is sterically hindered) and five of them are secondary OH groups. Taking into consideration this fact, the sucrose fulfills the requirement to be an appropriate chain extender/crosslinking agent, therefore, we selected sucrose as a chain extender/crosslinking agent to synthesize the target PURs.

Our purpose was to solve the challenge of the synthesis to fulfill the strict requirements of the different biological applications. Based on the strong, well established literature background, we accept that all ingredient used in this work are biocompatible and biodegradable. The biocompatibility and the biodegradation behaviors of both poly(*ε*-caprolactone) and certain poly(*ε*-caprolactone) containing polyurethanes are also extensively investigated and proved therefore we concentrated our efforts to the chemical solutions [12,13].

In this paper, we report a one pot synthesis and detailed characterization of biocompatible and completely biodegradable PUR networks synthesized from PCLD/HDI/sucrose systems.

## 2. Materials and Methods

### 2.1. Materials

Poly(ε-caprolactone) diol (PCLD, Mn = 2000 g/mol), dimethyl-sulfoxide (99.9 %, DMSO, stored on molecular sieve), 1,6-hexamethylene diisocyanate (HDI, reagent grade), Tin(II) 2-ethylhexanoate (reagent grade) were purchased from Sigma-Aldrich (Darmstadt, Germany) and were used as received. D(+)-sucrose (puriss, Ph. Eur. 6.) from Reanal (Budapest, Hungary) was powdered and dried in a vacuum oven at 40 °C for overnight before use. Toluene (analytical grade), acetone (99.9%, HPLC grade) from Sigma-Aldrich (Darmstadt, Germany), methanol (HPLC grade) from Merck (Darmstadt, Germany) and hexane (HPLC grade) from VWR (Debrecen, Hungary) were used without any purification.

### 2.2. Synthesis of the Prepolymer

The synthesis of prepolymer was basically carried out according to ref. [8] in melt. The modified method applied was the following: A 100 mL round-bottom, three-necked flask equipped with a mechanical stirrer, nitrogen inlet, reflux condenser and a nitrogen outlet was used. The temperature was kept at 90 °C by means of a silicon oil bath. Before assembly, all the glassware were dried at 140 °C for a day. The flask was charged with 20 g (10 mmol) of PCLD under continuous nitrogen flow. After complete melting of the PCLD the mechanical stirrer was turned on and 3.5 mL (22 mmol) HDI was introduced to the reactor. The homogeneous mixture was stirred at 80 °C for four hours. During the prepolymer synthesis only a negligible amount of gas formation may be observed. When the mixing time expired a sample (around 0.2 mL) was taken out from the reaction mixture for further analysis.

### 2.3. Synthesis of Sucrose Containing (SPUR) Polyurethane Networks

Predetermined amount of vacuum dried sucrose (9.5–0.95 mmol) was dissolved in 50 mL of anhydrous DMSO at 60 °C. After complete dissolution of sucrose, the solution was added in one portion to the melt prepolymer (synthesized according to Section 2.2.) during vigorous stirring (0.08 g, 0.2 mmol). Tin(II) 2-ethylhexanoate catalyst was also introduced to the reaction flask. The temperature was kept at 80 °C. The opaque mixture was then stirred until the mixture became homogenous again. It usually took place within a minute. To avoid the fast gelation the resulting viscous mixture was poured into Teflon coated pans within 2 min and cured at 60 °C for 24 h. The obtained SPUR sheets were dried in a vacuum oven at 40 °C for several (2–3) days for constant weight. During the synthesis no weight loss was observed.

### 2.4. Characterization

#### 2.4.1. Scanning Electron Microscopy (SEM)

To visualize the morphology of the SPUR samples scanning electron microscopy (SEM) was used. A secondary electron image was taken from the cut edge of SPUR samples after covering them by 30 nm conductive gold layer by a Hitachi S-4300 scanning electron microscope (Tokyo, Japan).

#### 2.4.2. Attenuated Total Reflectance Fourier-Transform Infrared (ATR-FTIR)

Attenuated total reflectance Fourier-transform infrared (ATR-FTIR) spectra were recorded on a PerkinElmer Spectrum Two Instrument equipped with Ultra ATR Two Sampling Accessory having a diamond-zinc-selenium composite prism (Waltham, MA, USA). The penetration depth of the IR beam into the SPUR sample is 6 µm. Thickness of the specimens were ca. 0.5 mm. Sixteen scans were taken. The spectra were evaluated by Spectrum ES 5.0 program.

#### 2.4.3. Swelling Experiments

SPUR samples (having initial measures: 10 × 15 × 0.2–0.4 mm, and initial weight: m_o_) were placed in 20 mL of different solvents in Erlenmeyer flasks of 50 mL at room temperature. The following solvents were used for swelling: acetone, DMSO, hexane, methanol, toluene and water. The swelled SPUR samples were taken out and the immersion fluid was wiped off from the surfaces with a paper towel. The weight gain was followed by an analytical balance in every hour. The weight gain was monitored until equilibrium was reached. It usually took around two days. The degree of swelling, i.e., the swelling ratio (%) (*Q*) is defined by Equation (1) as:

(1) $$Q=\frac{m-m_{o}}{m_{o}}\times 100$$

where m and m_o_ are the masses of the swollen and the initial (dry) sample, respectively.

After completing the swelling test, the absorbed solvent was removed in a vacuum oven at 40 °C until constant weight (m_1_). The gel content (*G%*) of the SPUR samples were calculated according to the following Equation (2):

(2) $$G(\%)=\frac{m_{1}}{m_{o}}\times 100$$

The crosslink density of SPUR samples was calculated according to the Flory-Rehner equation [14] as given by Equation (3):

(3) $$\nu_{e}=\frac{-\ln[(1-Y_{p})+Y_{p}+\chi Y_{p}^{2}]}{V_{s}(Y_{p}^{1/3}-\frac{Y_{p}}{2})}=\frac{\rho_{p}}{M_{c}}$$

where ν_e_ = crosslink density, Y_p_ = the volume fraction of the polymer, V_s_ is the molar volume of the solvent, χ is the polymer-solvent interaction parameter, ρ_p_ is the density of the polymer and M_c_ is the number average molecular weight between crosslink points. The polymer-solvent interaction parameter for the SPUR networks was determined as detailed in the results and discussion.

#### 2.4.4. Matrix-Assisted Laser Desorption/Ionization Time-of-Flight Mass Spectrometry (MALDI-TOF MS)

The Matrix-Assisted Laser Desorption/Ionization Time-of-Flight Mass Spectrometry (MALDI-TOF MS) measurements were carried out with a Bruker Autoflex Speed mass spectrometer equipped with a time-of-flight/time-of-flight (TOF/TOF) mass analyser. In all cases 19 kV acceleration voltage was used in the positive ion mode. To obtain appreciable resolution and mass accuracy ions were detected in the reflectron mode and 21 kV and 9.55 kV were applied as reflector voltage 1 and voltage 2, respectively. A solid phase laser (355 nm, ≥100 μJ/pulse) operating at 500 Hz was applied to produce laser desorption and 5000 shots were summed. The MALDI-TOF MS spectra were externally calibrated with poly(ethylene glycol) standard (M_n_ = 1540 g/mol).

Samples were withdrawn from the reaction mixture and dissolved in a mixture of THF and methanol (50/50 *v*/*v*) at a concentration of 10 mg/mL and allowed the free isocyanate groups to react with the methanol for 2 days. Samples for MALDI-TOF MS were prepared with 2,5-dihydroxy benzoic acid (DHB) matrix. The matrix was dissolved in a mixture of THF and methanol (50/50 *v*/*v*) at a concentration of 20 mg/mL. The matrix solution, the sample solution and sodium trifluoroacetate solution (5 mg/mL in THF/methanol (50/50 *v*/*v*)), used as the cationization agent to promote the ionization, were mixed in a 10:2:1 (*v*/*v*) ratio (matrix/analyte/cationization agent). A volume of 0.5 μL of the solution was deposited onto a metal sample plate and allowed to air-dry.

#### 2.4.5. Mechanical Tests

Instron 4302 type mechanical testing machine (Instron, Norwood, MA, USA), equipped with a 1 kN load cell, was used for tensile testing of the SPUR series. Four dumbbell specimens were cut (clamped length 60 mm) from the SPUR samples and the tensile was loaded at a strain rate 50 mm/min. For the data evaluation Instron series 9 Automated Materials Tester—version 8.30.00 software was used.

For the stress relaxation experiments an Instron 3366 (Instron, Norwood, MA, USA) type mechanical testing machine was used. The specimens were subjected to 100% elongation and the decay of the load at this strain was measured and evaluated under the supervision of the Instron Bluehill Universal V 4.05 (2017) software.

#### 2.4.6. Differential Scanning Calorimetry (DSC)

The thermal properties of the samples were evaluated by Differential Scanning Calorimetry (DSC) applying a DSC Q2000 power compensation equipment (TA Instruments, New Castle, DE, USA) operating at 10 °C/min heating rate. Nitrogen was used as protective atmosphere.

#### 2.4.7. Dynamic Mechanical Analysis (DMA)

The dynamic mechanical properties of the samples were carried out using Dynamic Mechanical Analysis (DMA) testing with a DMA Q800 device (TA Instruments, New Castle, DE, USA). The DMA traces were recorded in tension mode (dimension of the specimens: length: 25 mm, clamped length: 12 mm, width: 7 mm, thickness: ca. 0.5 mm) at an oscillation amplitude of 0.2% with a frequency of 1 Hz and applying a static load of 1 N. The temperature was varied between −100 °C and 200 °C with a heating rate of 3 °C/min.

## 3. Results and Discussion

Our purpose was to elaborate a one pot synthesis for the preparation of PCLD and sucrose containing polyurethane networks with regular structures and to improve their physical and chemical properties, such as swelling and flexibility for biological applications.

**Scheme 1:**
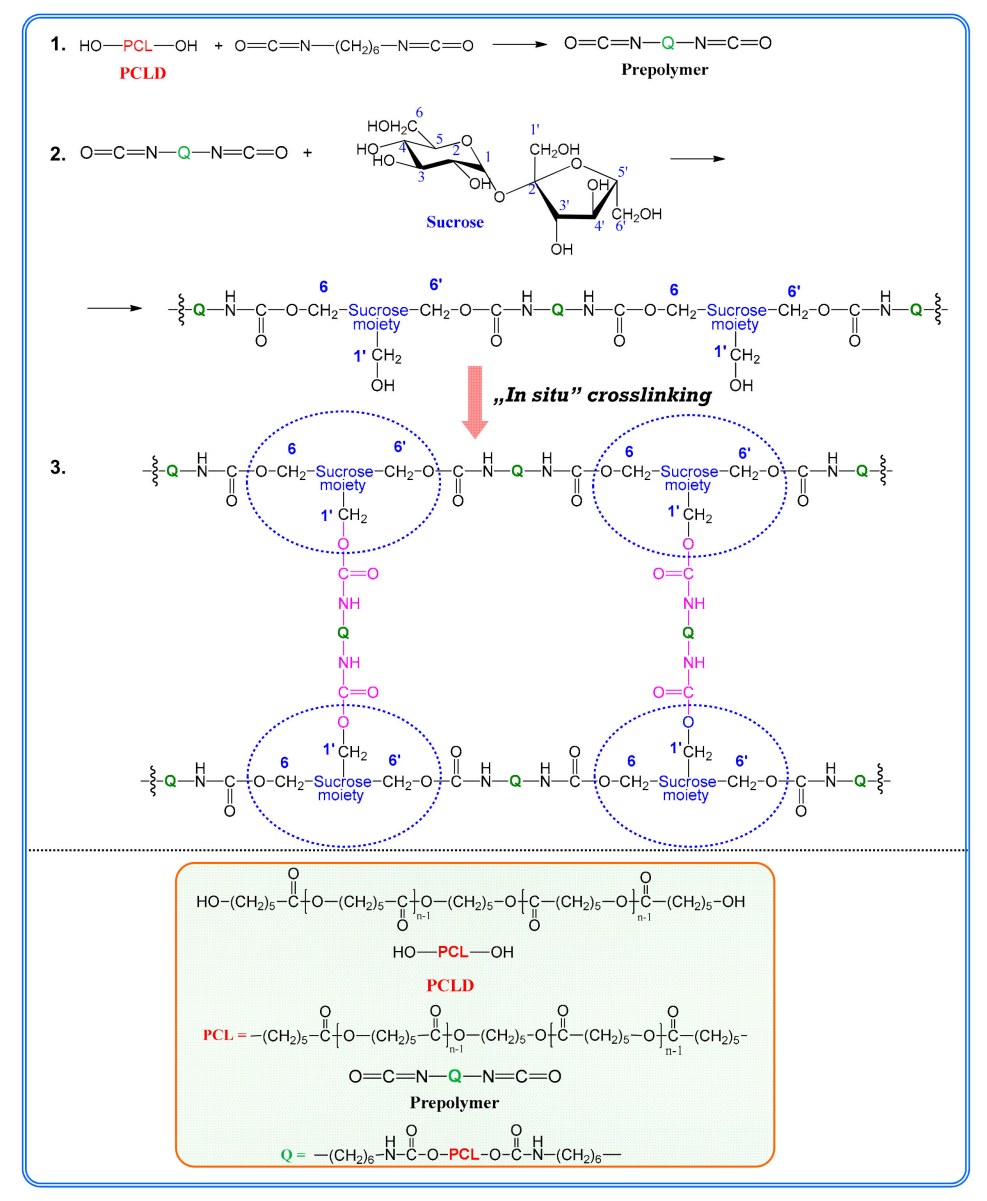
Synthesis path for the preparation of SPURs.

For the synthesis we worked out a novel synthetic strategy: first, a macro diisocyanates (prepolymer) was prepared from PCLD and HDI in melt. To obtain the main polymer chains of the network, sucrose as a multifunctional, small molecular weight chain extender/crosslinking agent dissolved in DMSO was used. The main polymer chains were thus formed by the polyaddition reaction between the prepolymer and the 6 and 6′ primary hydroxyl groups of sucrose (see Scheme 1).

The crosslinking reaction took place by the reaction of the excess of the free isocyanate groups in the prepolymer with the residual primary hydroxyl group, most probably, in the 1′position of sucrose. Thus, by varying the Mn-s of PCLD and the molar ratios of the prepolymer to sucrose, the distances between the main polymer chains and the crosslink density can also be altered, respectively (Scheme 1)

Based on the synthesis route presented in Scheme 1, a series of SPUR samples was prepared. The compositions, densities and Shore A hardness of SPUR samples are summarized in Table 1.

As can be seen in Table 1 the mol ratio of prepolymer and the sucrose was varied within one order of magnitude. The prepolymer concentration was kept constant while the concentration of sucrose in DMSO was changed gradually as shown in Table 1.

During the synthesis there was no weight loss, indicating that all reactants were built in the SPUR networks formed. This finding agrees well with the results from the swelling experiments (see later).

### 3.1. Infrared Spectroscopy of the SPUR Samples

**Figure 1:**
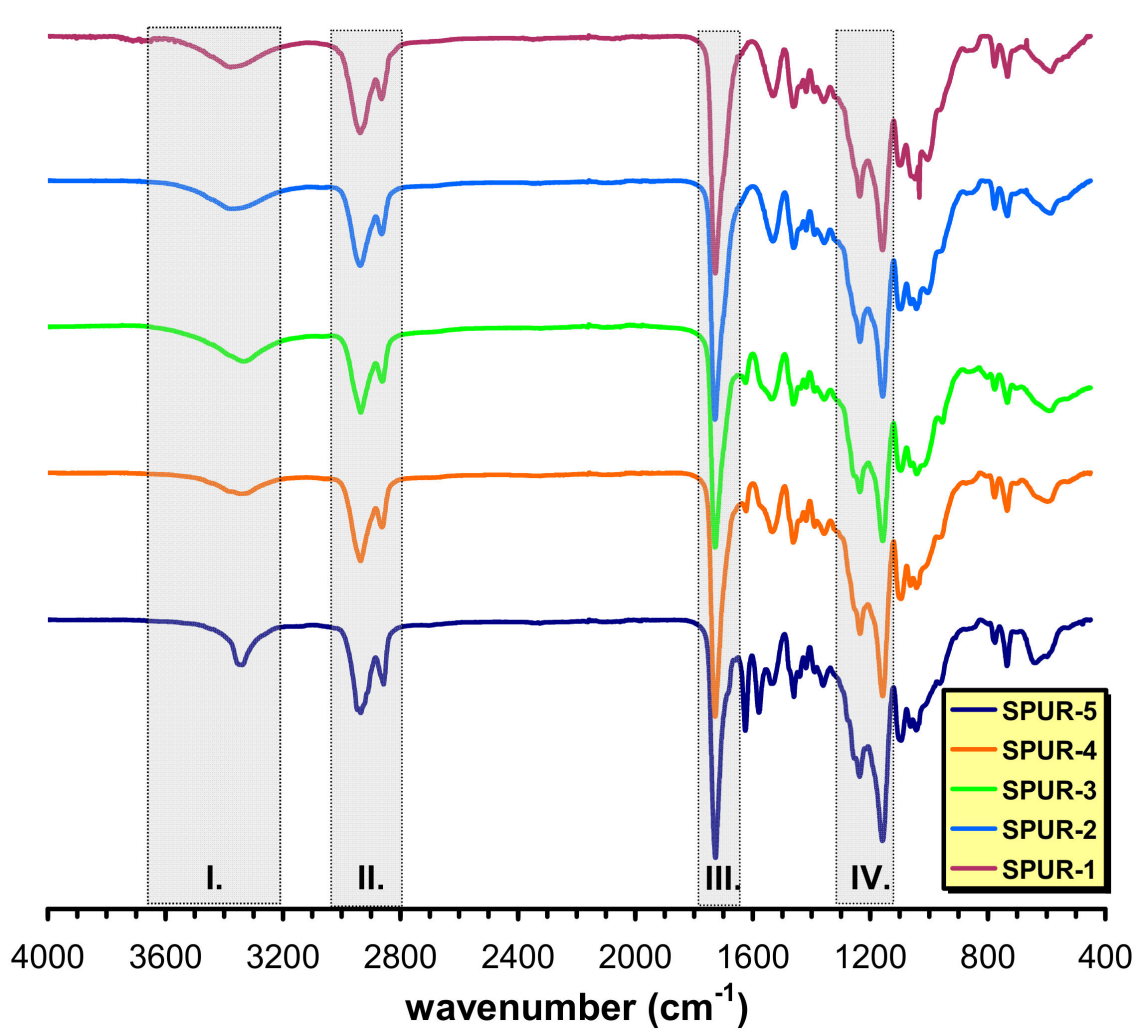
ATR-FTIR spectra of the SPUR samples.

In order to get information on the molecular structures of the SPUR networks formed Attenuated Total Reflectance Fourier-Transform Infrared (ATR-FTIR) spectra were recorded (Figure 1).

As seen in Figure 1, the intermolecularly bonded OH stretching, which might overlap with the NH stretching vibrations, especially at lower prepolymer/sucrose molar ratios, occur in the range of 3358–3334 cm^−1^ (shaded area I). A double band at 2937 and 2863 cm^−1^ (shaded area II) and at 1361 cm^−1^ belonging to CH_2_ stretching and bending vibrations, respectively can also be observed and a strong absorption band of C=O stretching is visible at 1727 cm^−1^ (shaded area III). An absorption band at 1537 cm^−1^ is attributed to the C-N stretching. The presence of this band in the ATR-FTIR spectrum confirms the formation of polyurethane linkages. The =C-O/-C-O-C- vibrations are assigned to appear in the range of 1150–1230 cm^−1^ (shaded area IV). In addition, no absorption can be seen at around 2275–2230 cm^−1^ (i.e., no absorption in the 2750–1850 cm^−1^ region), indicating no residual isocyanate groups present after 24 h of curing at 60 °C.

### 3.2. MALDI-TOF MS Investigations

**Figure 2:**
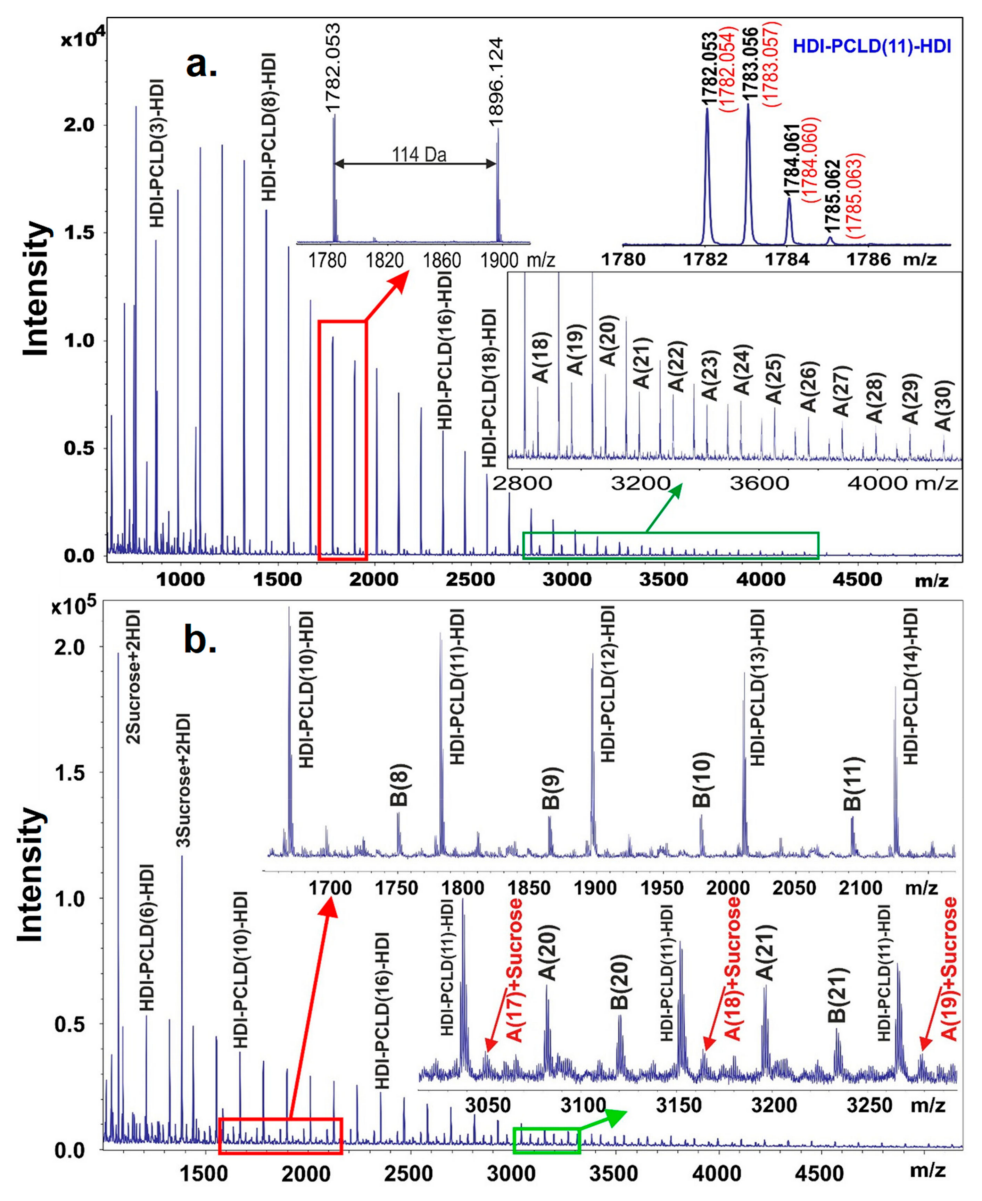
MALDI-TOF mass spectra of the samples obtained from the PCLD-HDI reaction mixture (Reaction conditions: temperature = 90 °C, reaction time: 4 h) (**a**) and from the HDI-PCLD prepolymer sucrose reaction (Reaction conditions: temperature = 90 °C, reaction time: 3 min, the molar ratio for PCLD/HDI/sucrose is 1/2.2/0.95, sample SPUR-1) (**b**). The numbers in the brackets represents the number of the caprolactone repeating unit. The isotopic distribution, the measured and the calculated (red in brackets) *m*/*z* values are shown in the inset (top right corner of Figure 2a).

The MALDI-TOF MS spectrum obtained on the prepolymer poly(ε-caprolactone)-diol (PCLD) with two HDI end-groups is shown in Figure 2. Prior to recording the MALDI-TOF mass spectrum the free isocyanate groups in the prepolymer were reacted with methanol in order to prevent further reaction of the free isocyanate groups with the matrix molecules. Furthermore, to enhance the cationization and to obtain mainly sodiated oligomers, sodium trifluoroacetate was added to the reaction mixture. As seen in Figure 2, the mass difference between the neighboring peaks is 114 Da that corresponds to the mass of a caprolactone repeat unit.

Furthermore, the measured masses match those of the PCLD with two HDI and two methanol units as expected for a prepolymer HDI-PCLD-HDI. For example, as illustrated in the top right corner of Figure 2a. inset, the measured monoisotopic mass at *m*/*z* 1782.053 is in line with that found for the composition [C_89_H_154_N_4_O_30_+Na]^+^ (*m*/*z* 1782.054). On the other hand, as seen in the zoomed MALDI-TOF MS spectrum (Figure 2a., in the range of *m*/*z* 2800–4000) in addition to the main series, series A corresponding to a HDI-PCLD-HDI-PCLD-HDI oligomer series also appeared.

In the next synthetic step the resulting prepolymer was further reacted with sucrose in DMSO. After a short reaction time, in order to avoid considerable cross-linking, a sample was withdrawn from the reaction mixture and analyzed by MALDI-TOF MS to prove that successful coupling reactions have been taken place between the prepolymer and sucrose. Indeed, as it turns out from Figure 2b, series B appeared in the MALDI-TOF MS spectrum, which was identified as HDI-PCLD-HDI-Sucrose oligomers. Furthermore, at higher masses, series A reacted with sucrose was also detected as demonstrated in the lower inset of Figure 2b. In the low mass region of the MALDI-TOF MS spectrum products resulting from the reaction of sucrose with HDI, i.e., sucrose + HDI containing no PCLD oligomer were also detected. This latter finding is very intriguing, since two sucrose + 2HDI and three sucrose + 2 HDI were also formed as seen in Figure 2b. Thus, the presence of these compounds in the reaction mixture indicates that albeit sucrose has eight OH groups to be reacted with the isocyanate, they are not equally reactive. Furthermore, it seems unambiguous from these findings that two of these OH groups are more reactive than the others thus yielding linear versions of sucrose + HDI oligomers at the early stage of the reaction. This finding has also consequence on the cross-linking process with sucrose that will be discussed later.

### 3.3. Swelling Experiments

**Figure 3:**
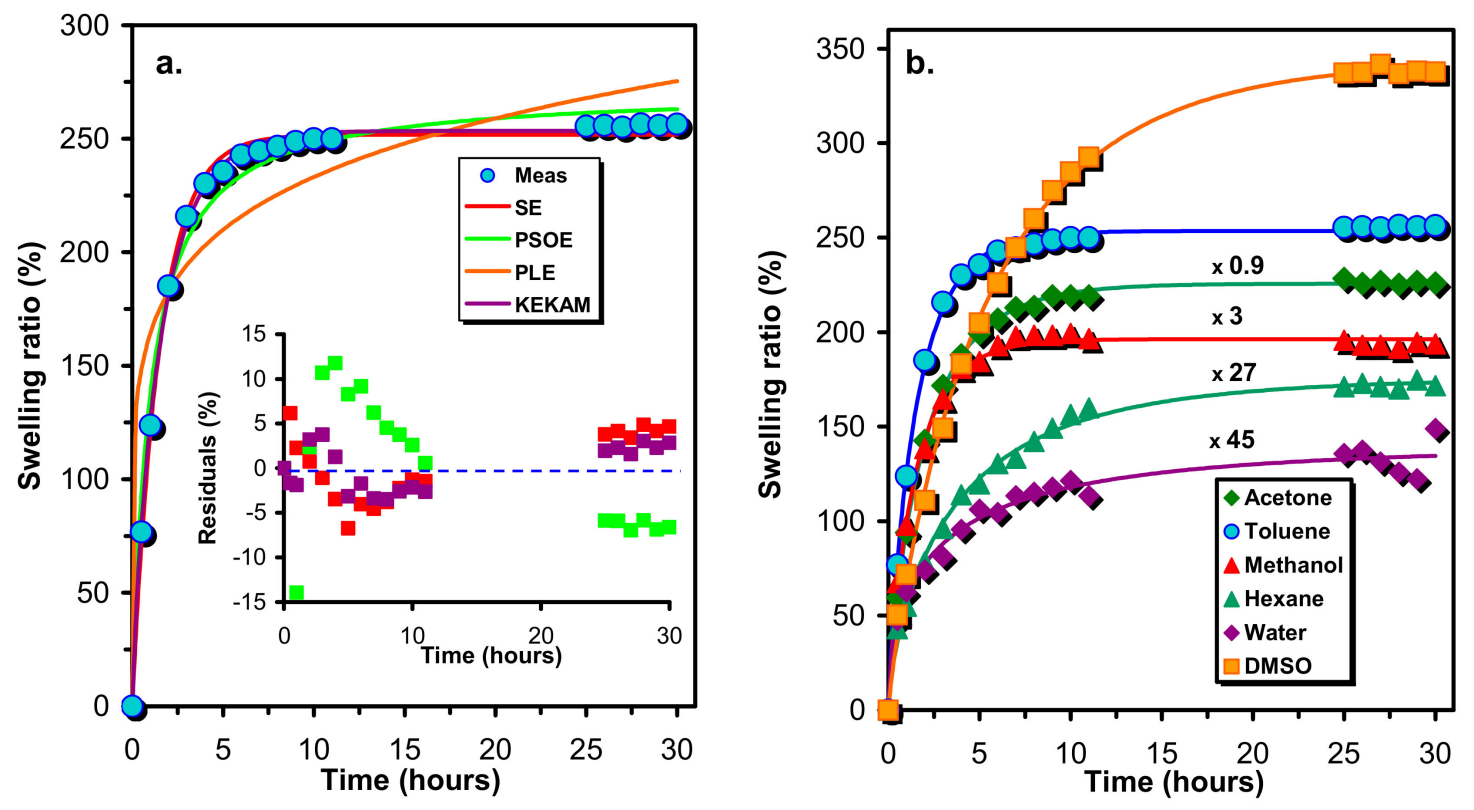
Variation of the swelling ratios with time for the sample SPUR-4 and fitting of the different models to the experimental data (solid lines) (a) and the dependence of the swelling ratio on the time together with the curves fitted by the streched exponential function (solid lines) (b). The inset in Figure 4a. shows the residuals as the difference between the experimental and the fitted data by the various models. The fitted parameters can be found in the Supplementary Materials (Table S1).

In order to gain insight into the crosslink densities and the nature of the network formed by the reaction of the prepolymer HDI-PCLD-HDI with sucrose, the resulting crosslinked polymers were swollen with solvents (such as n-hexane, toluene, acetone, methanol, DMSO and water) of different polarities and solubility parameters. The swelling of the SPUR samples was followed in time by measuring the weight of the swollen samples with respect to those of the dry ones and the swelling ratios (%) as a function of the time were plotted (Figure 3). To describe the swelling ratio (%) (Q) versus time curves various models were tried to fit to the experimental data. The investigated models included the single exponential (SE, Equation (4)), the integrated form of the pseudo second order rate equation (PSOE, Equation (5)) [15], the power law equation (PLE, Equation (6)) [16] and the stretched exponential function of Kolmogorov, Erofeev, Kozeeva, Avrami, Mampel (KEKAM, Equation (7)) [17].

(4) $$Q=A(1-e^{-kt})$$

(5) $$Q=\frac{kt}{A+kt}$$

(6) $$Q=kt^{\gamma }$$

(7) $$Q=A[1-e^{-(kt{)}^{\gamma }}]$$

where *k* and *A* are the rate coefficients and the equilibrium value of *Q* respectively, while *A* and γ stand for the constant of the stretched exponential function.

Figure 3a illustrates the results of fitting of Equations (4)–(7) to the experimental swelling ratios (%) versus time data obtained for the sample SPUR-4 and Figure 3a demonstrates the differences (residuals) between the fitted and the experimental data.

As seen in Figure 3a, the PLE model (Equation (6)) is unable to render the main characteristics of the experimental swelling ratio (%)-time dependences, whereas the other three models may give acceptable fitting. However, as it turns out from Figure 3a, KEKAM model (Equation (7)) provides the best fitting and this finding is also true for the rest of the SPUR samples investigated. Thus, the KEKAM model was applied for the description of the swelling properties of these polymer samples. As seen in Figure 3b, Equation (7) is indeed capable of the description of the variation of the swelling ratio with the time for various solvents. The parameters of the KEKAM model obtained by fitting it to the experimental data are compiled in Supplementary Table S1. According to the data of Table S1, the value of the parameter *γ* is around 0.8 independently of the solvent, while values of *k* are spannig from 0.16 to 1.65, however, no rigorous relationships can be established between these values and the degrees of crosslinks.

**Figure 4:**
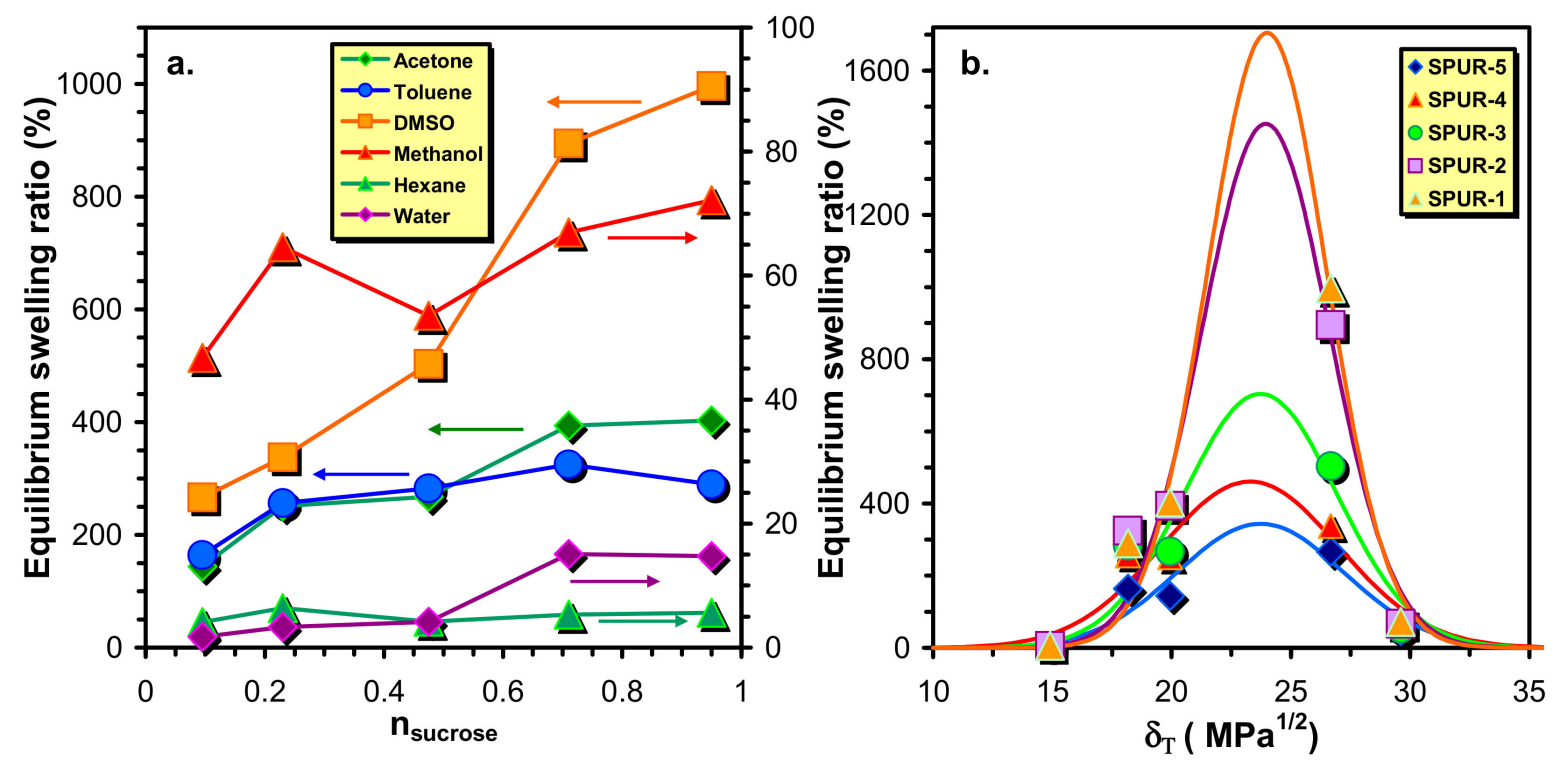
Variations of the swelling ratios with the molar ratios of sucrose (**a**) and the dependences of the swelling ratios on the values of the Hildebrand solubility parameter (*δ_T_*) (**b**). The solid lines in Figure 4b represent the fitted curves by Equation (8).

In Figure 4a the variations of the equilibrium swelling ratios (*Q_e_*) with the molar ratios of sucrose to that of prepolymer HDI-PCLD-HDI are plotted. As a general trend, it was found that the values of *Q_e_* decrease with the sucrose molar ratio from which it can be surmised that the crosslink densities also decrease with the increasing sucrose content. An explanation for this unusual finding will be given later. Furthermore, it is also evident from Figure 4a that the *Q_e_* –s at higher sugar content decrease in the order of DMSO > acetone > toluene > methanol > water > n-hexane, whereas at low sucrose content no considerable differences can be found in the *Q_e_* values of acetone and toluene.

To evaluate the dependence of the equilibrium swelling ratio on the solvent, the experimental equilibrium swelling ratios were plotted as a function of the solvent solubility parameters (*δ_T_* ) and Equation (8) [18] were fitted to these data to determine the solubility parameters of the crosslinked polymer networks (*δ_N_*).

(8) $$Q=Q_{e}e^{-\omega {\left(\delta_{T}-\delta_{N}\right)}^{2}}$$

where *Q_e_* is the equilibrium swelling ratio, and $\omega =\frac{\kappa {\bar{V}}_{1}}{RT}$, in which *κ*, ${\bar{V}}_{1}$, *R* and *T* are the empirical parameter, the molar volume of the solvent, the gas-constant and the temperature, respectively.

In Equation (8), parameters *Q_e_* at *δ =* *δ_T_,* *δ_N_* and *ω* were determined and the fitted curves by Equation (5) together with the corresponding experimental data are shown in Figure 4b and the fitted values of the parameters are compiled in Table 2.

It was found that the values of *δ_N_* fall into a narrow range with values of *δ_N_* = 23.3–24.0 (MPa)^1/2^, i.e., crosslinking in the applied extent does not alter significantly the solubility of the polymer network formed. In addition, we have also investigated the effect of the components of the Hansen solubility parameters [19], i.e., the dispersion *δ_D_*, the dipole *δ_P_* and hydrogen-bonded *δ_H_* solubility parameters on the swelling ratios. However, no significant correlation between the swelling ratios and the separated Hansen solubility parameters were found indicating that all of these three solubility parameters have an important role in determining the swelling properties of these polymer networks. It is to be noted that *δ_N_* values in Table 3. were used for the determination of crosslink densities. The crosslink densities were calculated using the Flory-Rehner equation (as indicated in the Materials and Methods). The results are compiled in Table 3.

**Figure 5:**
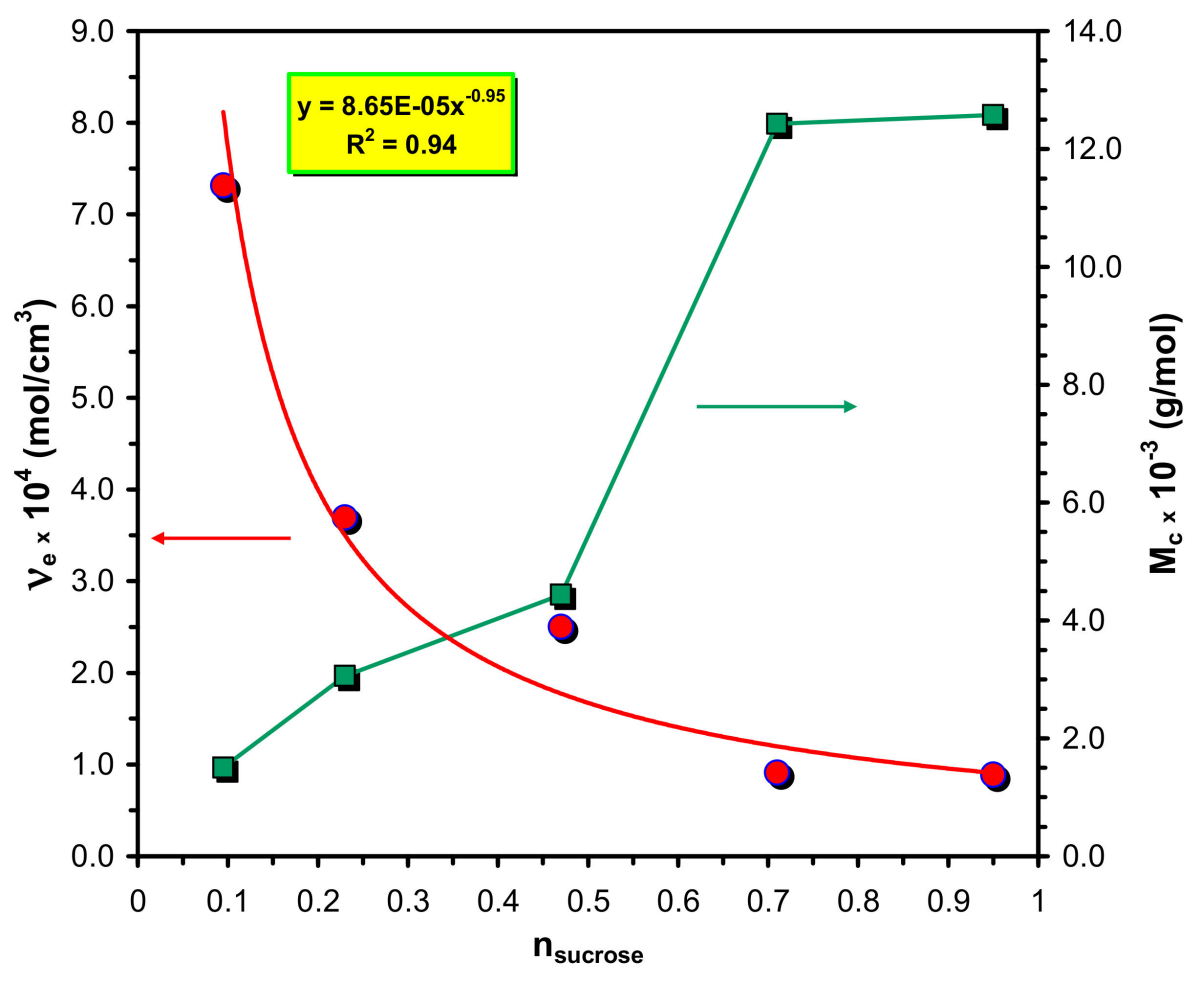
Dependence of the crosslink densities (*ν_e_*) and the average molecular weights of the segment between crosslinks (*M_c_*) on the sucrose content.

The graphical presentations of the data in Table 3. are shown in Figure 5.

According to the data of Table 3 the crosslink densities unexpectedly increase from 8.5 × 10^−5^ mol/cm^3^ to 7.3 × 10^−4^ mol/cm^3^ with the decreasing sucrose content and the dependence of *ν_e_* on the sucrose content can be described as $\nu_{e}=8.65\times 10^{-5}n_{\mathrm{sucrose}}^{-0.95}$ (see Figure 5), i.e., *ν_e_* is approximately inversely proportional to n_sucrose_. Furthermore, it also turns out from the M_c_ data in Table 3 that at the highest crosslink density M_c_ closely reflects to the M_n_ of the PCLD segment. To interpret this unique finding one should consider that there is a competitive reaction between the isocyanate groups of the prepolymer and the OH groups of sucrose. The sucrose bears eight free OH-groups, from which three are primary OH groups (one of them is sterically hindered) and five of them are secondary OH groups. The main polymer chains of the SPUR**s** are formed by the reaction of the prepolymer and the primary OH groups at the 6 and 6′ positions of sucrose. The crosslinking reaction between the main polymer chains may take place by the reaction of the free isocyanate groups of the prepolymer with the less reactive primary OH group at the 1′ position of sucrose to form the major skeleton of the SPUR networks (see Scheme 1). Moreover, upon depleting the primary OH groups, i.e., if the prepolymer concentration is significantly higher than that of the sucrose, which is the case for the samples SPUR-3, SPUR-4 and SPUR-5, the excess of prepolymer can react with the secondary OH groups of sucrose resulting in increasing crosslink densities.

### 3.4. Morphology of SPURs by SEM

**Figure 6:**
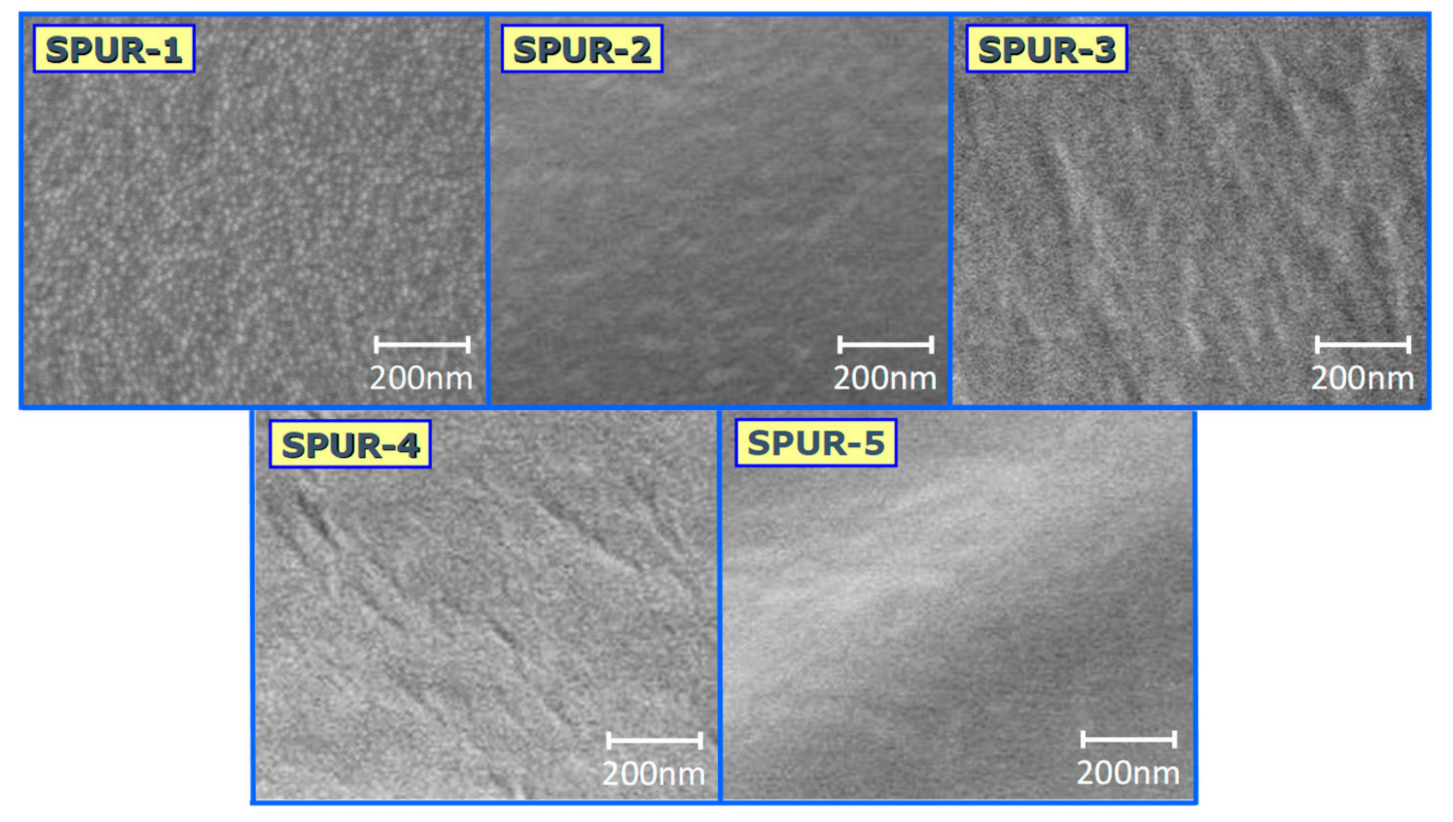
SEM images of SPUR samples.

The SEM images of SPUR samples are shown in Figure 6.

As can be seen in Figure 6, similar, but not identical microstructures were found. There are no deep inclusions and no significant phase separation occur. These facts suggest that the polymer networks obtained have regular structure.

### 3.5. Mechanical Properties of SPURs

The data in Table 4 shows the results of the uniaxial tensile measurements. All the polymers were very flexible and transparent before the tests.

As seen in Table 4, all the SPUR samples show low elastic modulus and high ultimate elongation values. Elastic (Young) moduli vary in the range of 2.1–5.3 MPa according to a minimum curve depending on the composition, i.e., the crosslink density.

However, the ultimate elongation data reveals an increasing trend with the decreasing amount of sucrose (i.e., with increasing crosslink density, see Table 3). On the other hand, more crosslink gives higher stress at break (sample SPUR-1 is an exception to that) and increasing ultimate elongation at the same time.

An attempt was made to describe the resulting stress-strain (*σ−ε*) curves at low and high strains. The description of the *σ−ε* relationship was based on the standard linear solid (SLS) viscoelastic model. As it was shown earlier in uniaxial tensile mode and at constant strain rate (d*ε*/dt) “the equation of motion” can be given by Equation (9) [20,21].

(9) $$\sigma +a_{1}\left(\frac{d\varepsilon }{dt}\right)\frac{d\sigma }{d\varepsilon }=a_{2}\varepsilon +a_{3}\frac{d\varepsilon }{dt}$$

where *a*_1_, *a*_2_ and *a*_3_ are the parameters that contain the moduli of springs and the viscosity of the dashpot in the constitutive SLS model.

Equation can be solved analytically to obtain Equation (10).

(10) $$\sigma =b_{1}[\varepsilon +b_{2}(1-e^{-b_{3}\varepsilon })]$$

where $b_{1}=a_{2}$, $b_{2}=(d\varepsilon /dt)(a_{3}/a_{2}-a_{1})$ and $b_{3}={\left[(d\varepsilon /dt)a_{1}\right]}^{-1}$

**Figure 7:**
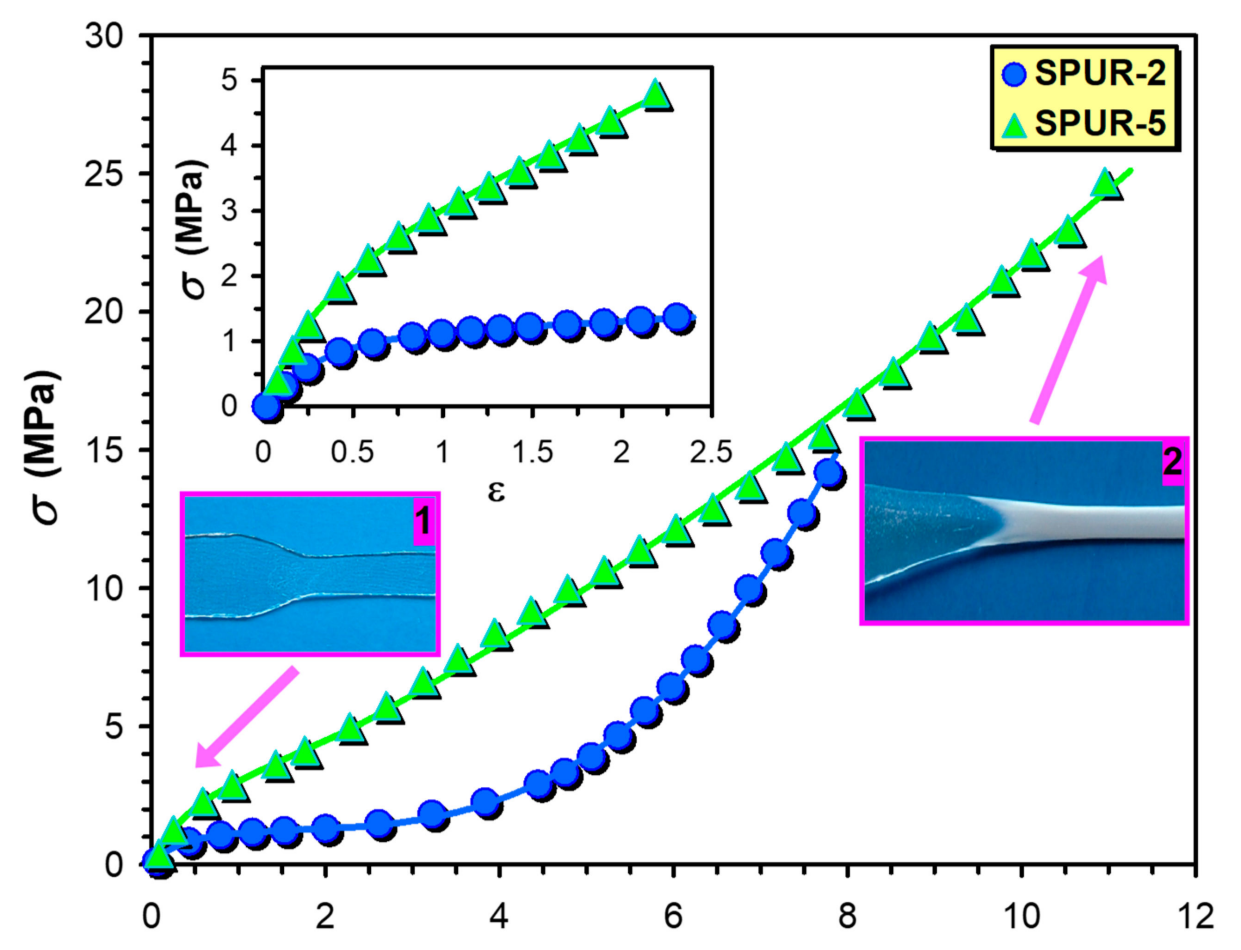
The stress-strain (*σ-ε*) curves for the samples SPUR-5 and SPUR-2. The symbols and the solid lines stand for the experimental data and the fitted curves, respectively. The inset figure reveals the lower part of the *σ−ε* curves up to ε = 2.2. The pictures illustrate the dumbbell from sample SPUR-5 at low (1) and at high strain (2). The fitted parameters for the sample SPUR-5 are b_1_ = 1.41 MPa, b_2_ = 1.18 and b3 = 3.24 (i.e., [(dε/dt)a_1_]^−1^ = 3.24, a_2_ = 1.41 MPa, (dε/dt)a_3_ = 2.10 MPa and α = 0.136 MPa and β = 0.75), while for the sample SPUR-2 these parameters are b_1_ = 0.12 MPa, b_2_ = 8.80 and b_3_ = 3.15 i.e., [(dε/dt)a_1_]^−1^ = 3.15, a_2_ = 0.12 MPa, (dε/dt)a_3_ = 1.09 MPa and α = 0.084 MPa and β = 1.75). (The stress-strain (*σ-ε*) curves for the samples SPUR-1, SPUR-3 and SPUR-4 are shown in Figures S1–S3, respectively in the Supplementary Materials).

The stress-strain (*σ* − *ε*) curves for the samples SPUR-5 and SPUR-2 are shown in Figure 7. and in the inset of Figure 7. As it turns out from the inset of Figure 7, Equation (9) is capable of rendering the experimental *σ-ε* curves up to moderate strains (*ε* < 2.2). However, it fails to describe the whole (*σ-ε*) curves especially at higher strains where marked deviations from the calculated ones have been observed. These deviations can be attributed to the upward curvature of the *σ-ε* plot, i.e., *σ* increases more rapidly with the increasing *ε* above ca. 2.2. These upward curvatures were supposed to be the effect of strain hardening, i.e., crystallization of the polymer segments can take place upon increasing strain, yielding an increase in the apparent modulus [20]. It is to be noted that a similar effect was observed and described for the epoxy-polyurethane (EPU) shape memory polymers [21]. The model that takes into account the strain hardening above a critical value of *ε* (*ε* > *ε*_L_) is similar to that reported for the EPUs, with an extension that the present model provides a more general description of strain-hardening by introducing a new variable (*β*) as seen in Equation (11).

(11) $$a_{2}(\varepsilon )=a_{2}+\alpha {\left(\varepsilon -\varepsilon_{L}\right)}^{\beta }\;(\mathrm{if}\;\varepsilon \;>\;\varepsilon_{L})$$

(12) $$d\sigma /d\varepsilon ={\left[(d\varepsilon /dt)a_{1}\right]}^{-1}[a_{2}(\varepsilon )\varepsilon +a_{3}(d\varepsilon /dt)-\sigma ]$$

Integrating Equation (12) numerically (after the substitution of Equation (11) into Equation (12) and the fitted values obtained by Equation (10)) and then fitting it to the experimental data over the whole *ε* range, the values of *α* and *β* can now be determined. One can also realize that the fitted (solid lines) and the experimental (symbols) values are in good agreement (see Figure 7).

It is interesting to note that *σ* increases more rapidly with *ε* in the case of sample SPUR-2 than it does for the sample SPUR-5 and this finding is also reflected in the values of *β* which are 0.75 and 1.75 for the sample SPUR-5 and SPUR-2, respectively. Furthermore, as seen in Figure 7, the stress at break for the sample SPUR-2 is lower than for the sample SPUR-5. These findings are in line with the crosslink densities that are 9.1 × 10^−5^ and 7.3 × 10^−4^ mol/cm^3^ for sample SPUR-2 and SPUR-5, respectively. At lower crosslink densities there are larger free spaces available for the polymer chains to crystallize giving rise to a steeper increase in *σ* with *ε*, and providing lower stress at break at the same time.

In addition, as illustrated by Figure 7, at low strains the sample is transparent (1), whereas at high strain it becomes opaque (white) due to the crystallization of the polymer segments upon the effect of strain (2), which also supports the validity of our approach.

**Figure 8:**
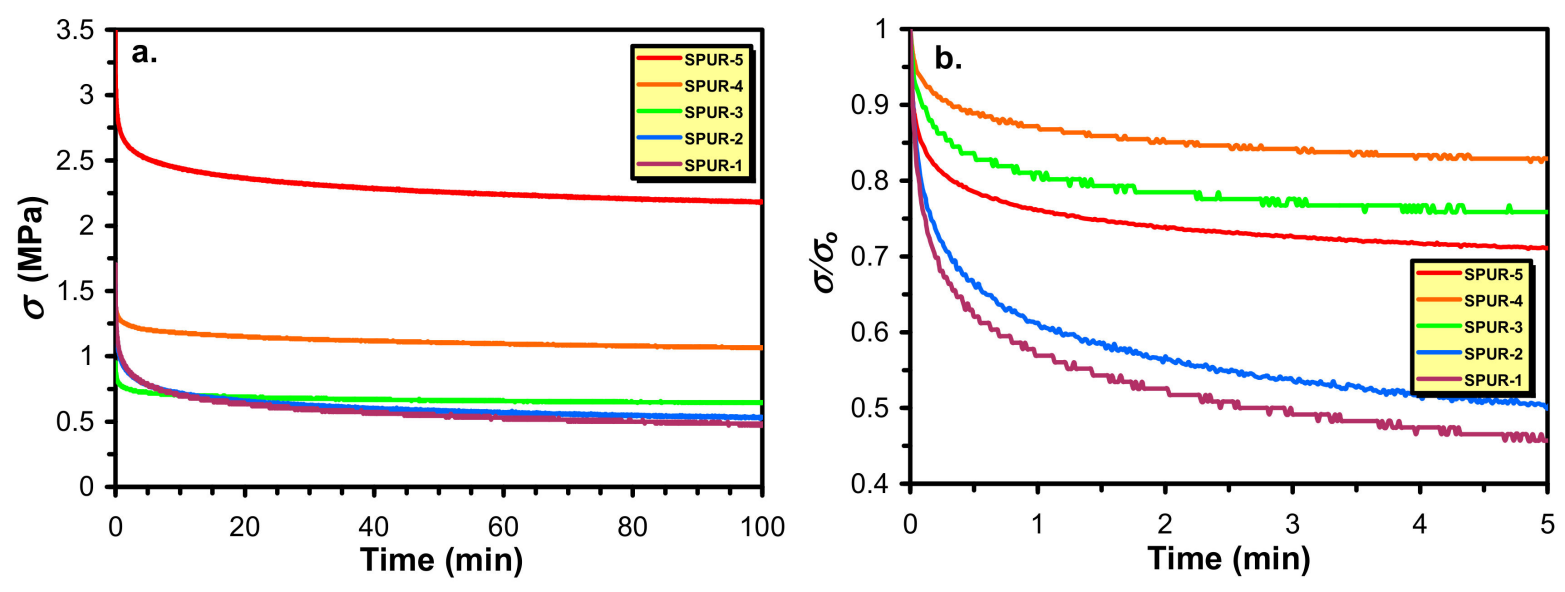
Stress (σ) (**a**) and relative stress (σ/σ_o_) (**b**) relaxation curves for the SPUR samples. The relative stress is defined as σ/σ_o_, where σ_o_ and σ are the initial and the instant stress, respectively.

In addition to these mechanical investigations, stress relaxation experiments were also performed to get deeper insight into the relaxation processes of these SPUR samples and in parallel, to determine the crosslink densities. The stress and the relative stress relaxation curves are demonstrated in Figure 8.

As it turns out from Figure 8a there is a relative fast relaxation process at the beginning of the relaxation curves that is followed by a slower relaxation period and eventually reaching a plateau value of *σ* that corresponds to the equilibrium relaxation modulus. Furthermore, as it is also seen from Figure 8b, the extent of decrease in the values of *σ/σ_o_* with time and to the equilibrium value follows an order of SPUR-4 > SPUR-3 > SPUR-5 > SPUR-2 > SPUR-1. This trend is almost in line with the decreasing crosslink densities for these SPURs except sample SPUR-5. This exception may highlight the fact that although the relative “viscous” part (relaxation to the equilibrium state) is mainly determined by the crosslink densities other factors can also affect the relaxation process. Moreover, the equilibrium stress value called as equilibrium modulus (*σ_eq_*) is directly related to the crosslink density of elastomers (*ν_r_*) according to theory of rubbers [22] as shown by Equation (13).

(13) $$\nu_{r}=\frac{\sigma_{eq}}{RT(\lambda -1/\lambda^{2})}$$

where *λ*, *R* and *T* are the extension ratio (i.e. the ratio of the extended and the initial length of the sample), the universal gas-constant and the temperature in Kelvin, respectively.

The corresponding crosslink densities calculated by Equation (13) are compiled in Table 5.

Comparing the data of Table 5 with that of Table 3, one can realize that the crosslink densities determined by the two methods (swelling and stress relaxation) agree relatively well. Hence, the decreasing crosslink densities with the increasing sucrose content is again supported by an independent method.

### 3.6. Thermal and Thermomechanical Properties of PURs

**Figure 9:**
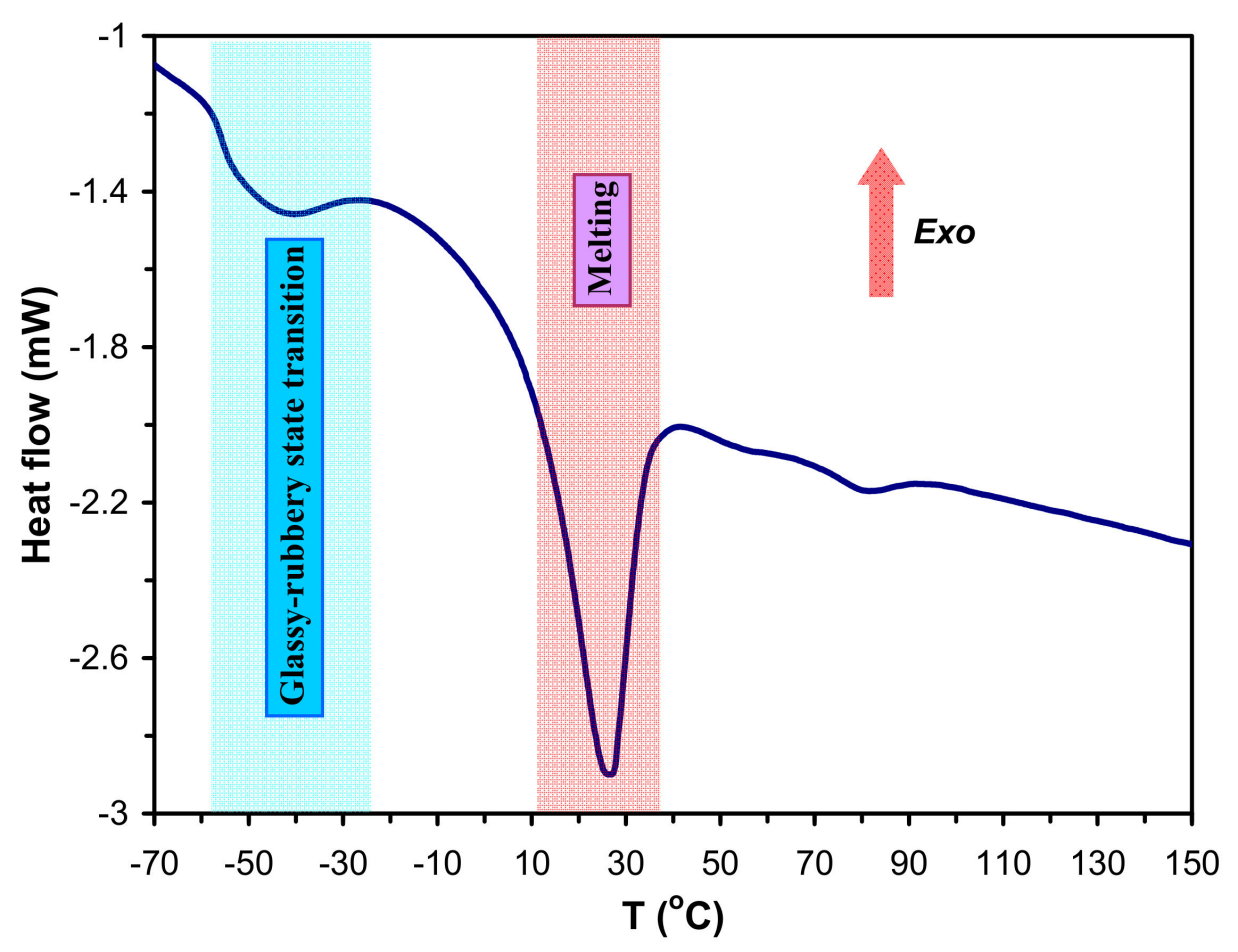
Representative DSC trace for the sample SPUR-3 (T_g_ = −56 °C, and T_m_ = 26 °C) (The DSC traces for the samples SPUR-1, SPUR-2, SPUR-4 and SPUR-5 can be seen in Figures S4–S7, respectively in the Supplementary Materials).

The thermal properties of the SPUR samples were investigated by differential scanning calorimetry (DSC) to determine their thermal transitions such as the glass transition temperature (T_g_) and the melting temperature (T_m_). A typical DSC trace for the sample SPUR-3 along with the characteristic thermal transitions are shown in Figure 9.

As seen in Figure 9 the T_g_ occurs between −50°C and −60 °C, while T_m_ are present at 20–30 °C. The values of T_g_ and T_m_ of SPUR samples determined from DSC measurements are presented in Table 6**.**

As it turns out from Table 6, the T_g_-s of the SPUR samples vary from −58 °C to −51 °C with the decreasing sucrose content, i.e., T_g_ increase with the increasing crosslink density. This finding is in line with the fact that chain mobility is gradually reduced with the increasing crosslink densities resulting in an increasing trend for T_g_ values. Furthermore, it can also be expected that at higher crosslink densities the crystallization is more hindered giving rise to lower T_m_ values. On the other hand, the T_m_ values for the PCLD segments in the SPUR samples are close to the room temperature, and thus are much lower than that of the high molecular weight poly(ε-caprolactone) (PCL), which melts at ca. 60 °C. That means the PCLD segments in the SPUR networks are in amorphous rubbery physical state. This fact is highly favorable for improving the chemical and/or biodegradation process in biological environments.

**Figure 10:**
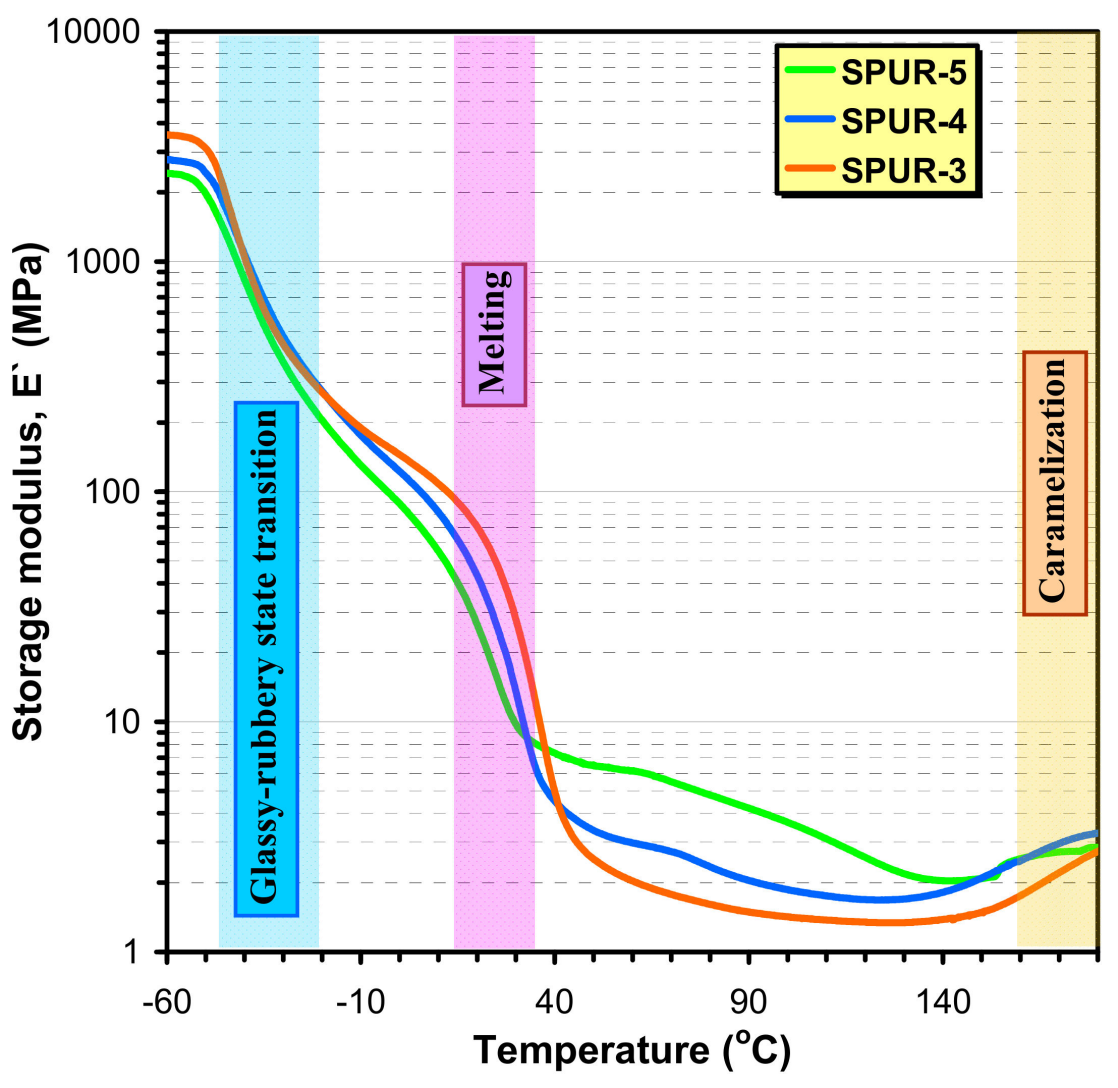
Variation of the storage modulus (E′) with the temperature for the samples SPUR-5, SPUR-4 and SPUR-3. (The DMA traces for the samples SPUR-1 and SPUR-2 are shown in Figures S8 and S9, respectively in the Supplementary Materials).

The dynamical mechanical properties of SPUR samples were also characterized by DMA. The dependences of the storage moduli (E′) on the temperature for the samples SPUR-5, SPUR-4 and SPUR-3 are shown in Figure 10.

As seen in Figure 10 different regions can be distinguished on the DMA curves: at low temperature (at about −50 °C) the considerable decrease in E′ can be attributed to the transition from the glassy to the rubbery state and a further, marked decrease at around 20–30 °C is due to the melting of the crystalline segments. The temperatures at which these processes occur are in good agreement with the values of T_g_ and T_m_ obtained from DSC measurements (see Table 6). At higher temperatures, however, the melting is followed by a gradual loosening of the physical network, but owing to the chemical crosslinks, DMA curves exhibit rubbery like plateaus in the temperature range of 100–150 °C. Typically, above 140 °C, an increase in the storage modulus with the temperature can be observed. This finding is most probably due to the caramelization of the sucrose and thus this process leads to the formation of tighter crosslinks between these sucrose moieties. Furthermore, it can also be surmised that higher crosslink densities yield lower T_m_-s owing to the reasons outlined above. Indeed, as seen in Figure 10. the melting of the crystalline phase occurs in the order of T_m_: SPUR-5 < SPUR-4 < SPUR-3 and this order is in line with the crosslink densities determined from swelling and the stress relaxation experiments (see Table 3 and Table 5) as well as with the data of Table 6. In addition, the rubbery-like plateau provides another way to estimate the crosslink density (*ν_d_*) from DMA results according to Equation (14).

(14) $$\nu_{d}=\frac{E\prime }{3RT}$$

where *E′* is the value of the storage modulus at the plateau value of *T* and *R* is the universal gas-constant, and *T* is the temperature in Kelvin.

The crosslink densities from DMA data for samples SPUR-5, SPUR-4, SPUR-3, SPUR-2 and SPUR-1 were determined to be 2.1 × 10^−4^ mol/cm^3^, 1.7 × 10^−4^ mol/cm^3^, 1.3 × 10^−4^ mol/cm^3^, 8.8 × 10^−5^ mol/cm^3^ and 8.6 × 10^−5^ mol/cm^3^, respectively. Although these values are lower than those determined by swelling and stress relaxation experiments the order of *ν_d_* agrees well.

## 4. Conclusions

For improving the mechanical, chemical and biological properties of poly(ε-caprolactone) for potential biomedical and pharmaceutical applications novel polyurethanes were synthesized involving sucrose as chain extender/crosslinking agent and HDI. For the selection of the reactants and solvents the proposals of European Pharmacopoeia-8 were considered, i.e., no aromatic diisocyanates were used and the solvents applied were selected from class 2 and class 3 groups.

For the synthesis, a novel synthetic strategy was elaborated and used. First a prepolymer was synthesized in melt from PCLD and HDI. The main polymer chains of the networks were prepared “in situ” by reacting the prepolymer with the two most reactive primary hydroxyl groups at the 6 and 6′ position of the sucrose molecule. The networks were formed after reacting the sucrose less reactive primary OH (-OH group at 1′ position) with the excess of the prepolymer. This way, the distances between the main polymer chains can be changed deliberately by varying the M_n_-s of the prepolymer.

The crosslink density can simply be adjusted with the molar ratio of the prepolymer and the sucrose. The prepared rubbery sheets showed highly improved swelling properties. Furthermore, the mechanical properties such as the tensile behaviors and flexibility of SPURs can be chemically tailored.

## Figures and Tables

**Table 1:** Sample name, composition, density and Shore A hardness of sucrose containing PUR polymers (SPUR samples).

Sample Name	Composition (mol Ratio) PCLD/HDI/Sucrose	Sucrose % (m/m)	Density (g/cm^3^)	Hardness Shore A Scale (%)
SPUR-1	1/2.2/0.95	12.1	1.136	67
SPUR-2	1/2.2/0.71	9.3	1.133	62
SPUR-3	1/2.2/0.475	6.4	1.109	51
SPUR-4	1/2.2/0.238	3.3	1.124	52
SPUR-5	1/2.2/0.095	1.4	1.102	66

**Table 2:** The values of parameters *Q_e_*, *ω* and *δ_N_* obtained by fitting of Equation (8) to the corresponding experimental data.

Sample	*Q_e_* (%)	*ω* (MPa^−1^)	*δ_N_* (MPa^1/2^)
SPUR-1	1705 ± 540	0.078 ± 0.026	24.0 ± 0.3
SPUR-2	1452 ± 496	0.069 ± 0.027	23.9 ± 0.4
SPUR-3	705 ± 253	0.049 ± 0.024	23.7 ± 0.5
SPUR-4	460 ± 126	0.036 ± 0.016	23.3 ± 0.5
SPUR-5	344 ± 113	0.041 ± 0.019	23.7 ± 0.6

**Table 3:** The gel content (*G(%*)), crosslink densities (*ν_e_*) and the average molecular weights of segment between crosslinks (*M_c_*) with different sucrose content determined in DMSO.

Sample	*G*(%)	*ν_e_* × 10^4^ (mol/cm^3^)	*M_c_* × 10^3^ (g/mol)
SPUR-1	91.0	0.89	12.6
SPUR-2	81.8	0.91	12.4
SPUR-3	90.6	2.5	4.4
SPUR-4	98.3	3.7	3.1
SPUR-5	95.1	7.3	1.5

**Table 4:** Uniaxial tensile mechanical properties of SPURs.

Sample	Elastic Modulus (MPa)	Ultimate Elongation (%)	Stress at Break (MPa)
SPUR-1	5.0 ± 0.4	810 ± 110	22.6 ± 2.2
SPUR-2	3.3 ± 0.8	800 ± 40	14.8 ± 6.4
SPUR-3	2.1 ± 0.3	940 ± 330	15.8 ± 1.0
SPUR-4	2.8 ± 0.3	945 ± 110	24.3 ± 4.7
SPUR-5	5.3 ± 0.6	1100 ± 80	26.3 ± 1.3

**Table 5:** The crosslink densities calculated by Equation (13). for the SPUR samples.

Sample	ν_r_ × 10^4^ (mol/cm^3^)
SPUR-1	1.1
SPUR-2	1.2
SPUR-3	1.5
SPUR-4	2.4
SPUR-5	5.0

**Table 6:** The glass-transition (T_g_) and the melting temperature (T_g_) determined from DSC measurements.

Sample	T_g_ (°C)	T_m_ (°C)
SPUR-1	−58	27
SPUR-2	−58	27
SPUR-3	−56	26
SPUR-4	−52	30
SPUR-5	−51	23

## Supplementary Materials

The following are available online at https://www.mdpi.com/2073-4360/11/5/825/s1, Table S1. The A, k, and γ parameters obtained by fitting of the KEKAM model to the experimental data. Figure S1: The stress-strain (σ−ε) curve for the SPUR-1 sample. Figure S2: The stress-strain (σ−ε) curve for the SPUR-3 sample. Figure S3: The stress-strain (σ−ε) curve for the SPUR-4 sample. Figure S4: DSC trace for the SPUR-1 sample (Tg = −58 °C, and Tm = 27 °C). Figure S5: DSC trace for the SPUR-2 sample (Tg = −58 °C, and Tm = 27 °C). Figure S6: DSC trace for the SPUR-4 sample (Tg = −52 °C, and Tm = 30 °C). Figure S7: DSC trace for the SPUR-5 sample (Tg = −51 °C, and Tm = 23 °C). Figure S8: DMA trace (variation of the storage modulus (E′) with the temperature in the range of −60–+180 °C) for the SPUR-1 sample. Figure S9: DMA trace (variation of the storage modulus (E′) with the temperature in the range of −60–+180 °C) for the SPUR-2 sample.
