# Supplementary material for: Designed Polyurethanes for Potential Biomedical and Pharmaceutical Applications: Novel Synthetic Strategy for Preparing Sucrose Containing Biocompatible and Biodegradable Polyurethane Networks
Source: Polymers (Basel). 2019 May 7;11(5):825. doi: 10.3390/polym11050825 (PMC6587335; doi:10.3390/polym11050825)
Supplement: Supplementary file 1 [file polymers-11-00825-s001.pdf]

# Supplementary Materials for

## Designed Polyurethanes for Potential Biomedical and Pharmaceutical Applications: Novel Synthetic Strategy for Preparing Sucrose Containing Biocompatible and Biodegradable Polyurethane Networks

**Lajos Nagy[1], Miklós Nagy[1], Bence Vadkerti[1], Lajos Daróczi[2], György Deák[1], Miklós Zsuga[1], Sándor Kéki[1*]**

[1]Department of Applied Chemistry, Faculty of Science and Technology, University of Debrecen, Egyetem tér 1, H-4032 Debrecen,. Hungary;
nagy.lajos@science.unideb.hu (L.N.); miklos.nagy@science.unideb.hu (M.N.);
bencevadkerti94@gmail.com (B.V.); deak.gyorgy@science.unideb.hu (G.D.);
zsuga.miklos@science.unideb.hu (M.Z.)
[2]Department of Solid State Physics, Faculty of Science and Technology, University of Debrecen, Bem tér 18/b, H-4026 Debrecen,. Hungary
daroczi.lajos@science.unideb.hu (L.D.);
[*]Correspondence: keki.sandor@science.unideb.hu (S.K.); Tel: +36-52-512-900 (ext. 22455)

## Table of Contents

**Table S1.** The $A$, $k$, and $\gamma$ parameters obtained by fitting of the KEKAM model to the experimental data. ....................2
**Figure S1**: The stress-strain ($\sigma$–$\varepsilon$) curve for the SPUR-1 sample....................3
**Figure S2**: The stress-strain ($\sigma$–$\varepsilon$) curve for the SPUR-3 sample....................3
**Figure S3**: The stress-strain ($\sigma$–$\varepsilon$) curve for the SPUR-4 sample....................4
**Figure S4**: DSC trace for the SPUR-1 sample ($T_g$ = -58 °C, and $T_m$ = 27 °C)....................4
**Figure S5**: DSC trace for the SPUR-2 sample ($T_g$ = -58 °C, and $T_m$ = 27 °C)....................5
**Figure S6**: DSC trace for the SPUR-4 sample ($T_g$ = -52 °C, and $T_m$ = 30 °C)....................5
**Figure S7**: DSC trace for the SPUR-5 sample ($T_g$ = -51 °C, and $T_m$ = 23 °C)....................6
**Figure S8**: DMA trace (variation of the storage modulus (E`) with the temperature in the range of -60 - +180 °C) for the SPUR-1 sample ....................6
**Figure S9**: DMA trace (variation of the storage modulus (E`) with the temperature in the range of -60 - +180 °C) for the SPUR-2 sample ....................7

**Table S1.** The $A$, $k$, and $\gamma$ parameters obtained by fitting of the KEKAM model to the experimental data.

| Sample | | n-Hexane | Toluene | Acetone | DMSO | Methanol | Water |
|---|---|---|---|---|---|---|---|
| | $A$ = | 5.6(0.1) | 286(4) | 397(4) | 1040(9) | 78(1) | 15(7) |
| **SPUR-1** | $k$= | 0.80(0.03) | 0.70(0.04) | 0.39(0.01) | 0.15(0.01) | 1.65(0.17) | 0.07(0.04) |
| | $\gamma$ = | 0.99(0.05) | 0.61(0.03) | 0.76(0.02) | 0.87(0.01) | 0.91(0.18) | 0.44(0.1) |
| | $A$ = | 6.3(0.3) | 310(5) | 396(1) | 963(12) | 72(0.8) | -* |
| **SPUR-2** | $k$= | 0.22(0.03) | 0.36(0.03) | 0.96(0.02) | 0.16(0.01) | 0.88(0.07) | - |
| | $\gamma$ = | 0.93(0.20) | 0.47(0.03) | 0.80(0.03) | 0.99(0.06) | 0.93(0.10) | - |
| | $A$ = | -* | 268(2) | 251(7) | 503(19) | 50(1) | -* |
| **SPUR-3** | $k$= | - | 0.28(0.01) | 0.27(0.02) | 0.10(0.01) | 0.35(0.01) | - |
| | $\gamma$ = | - | 0.84(0.01) | 0.74(0.04) | 0.81(0.03) | 0.81(0.01) | - |
| | $A$ = | 6(1) | 254(1) | 250(1) | 344(2) | 66(1) | 3(1) |
| **SPUR-4** | $k$= | 0.25(0.01) | 0.65(0.01) | 0.49(0.01) | 0.18(0.01) | 0.68(0.02) | 0.33(0.06) |
| | $\gamma$ = | 0.72(0.03) | 0.89(0.02) | 0.84(0.01) | 0.88(0.02) | 0.88(0.40) | 0.52(0.07) |
| | $A$ = | 4(1) | 156(1) | 140(1) | 268(2) | 46(1) | -* |
| **SPUR-5** | $k$= | 0.16(0.01) | 0.80(0.05) | 0.80(0.06) | 0.88(0.01) | 0.52(0.01) | - |
| | $\gamma$ = | 1.03(0.07) | 0.76(0.05) | 0.63(0.04) | 0.82(0.01) | 0.82(0.02) | - |

*The swelling ratio in these cases were so low that the model could not be fitted unambiguously.

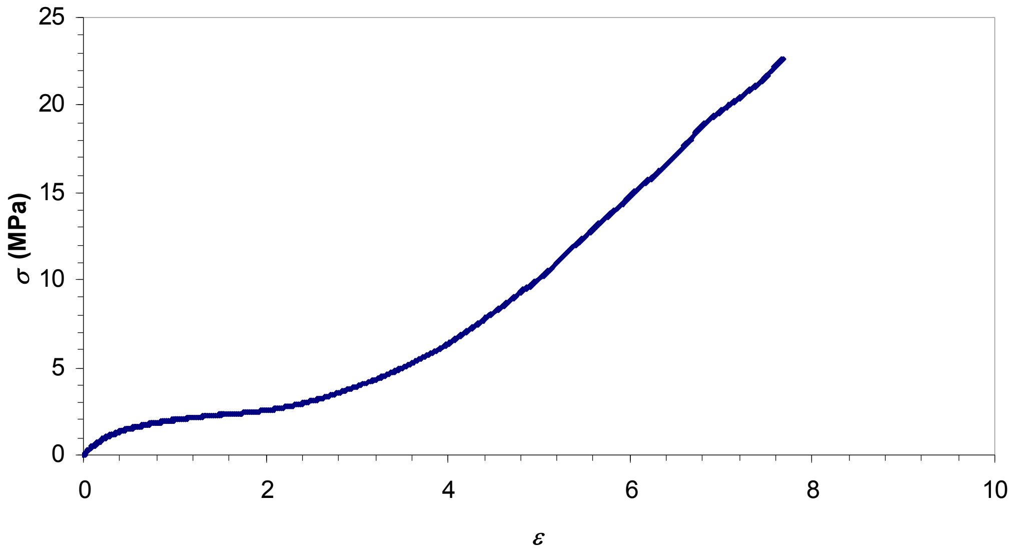


**Figure S1:** The stress-strain ($\sigma$–$\varepsilon$) curve for the SPUR-1 sample

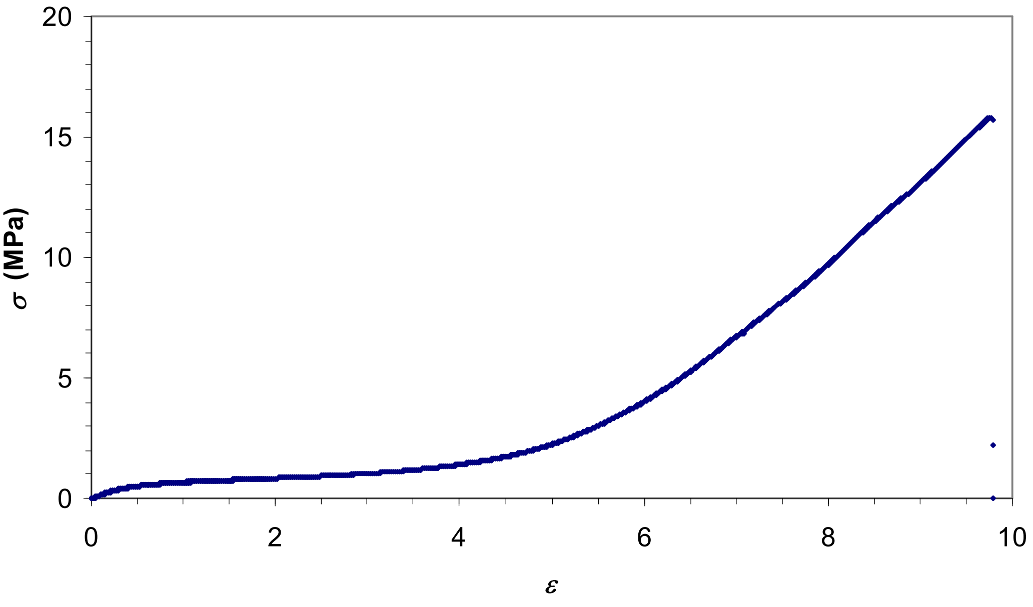


**Figure S2:** The stress-strain ($\sigma$–$\varepsilon$) curve for the SPUR-3 sample

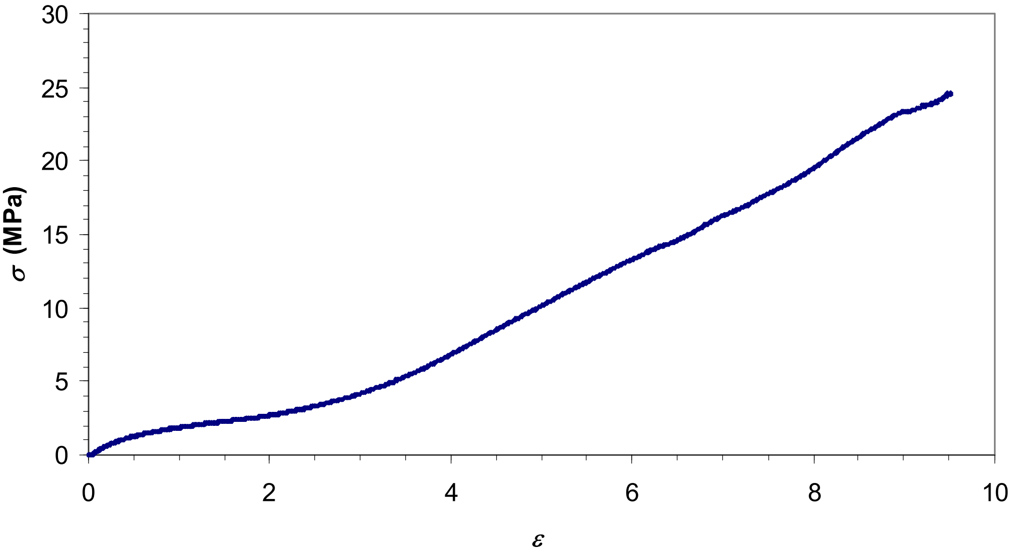


**Figure S3:** The stress-strain ($\sigma$–$\varepsilon$) curve for the SPUR-4 sample

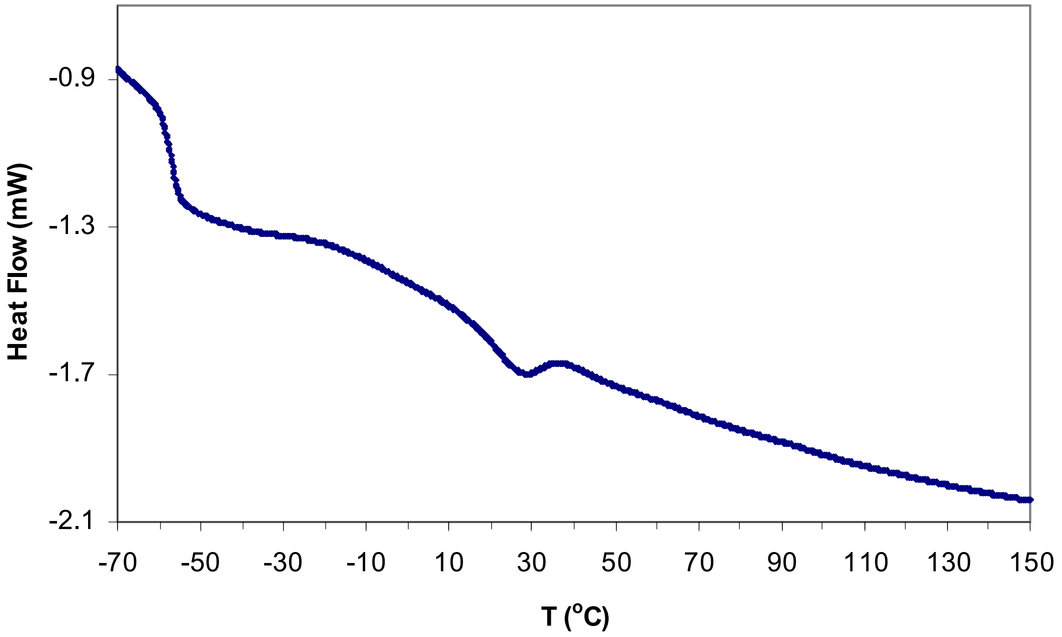


**Figure S4:** DSC trace for the SPUR-1 sample ($T_g$ = -58 $^oC$, and $T_m$ = 27 $^oC$)

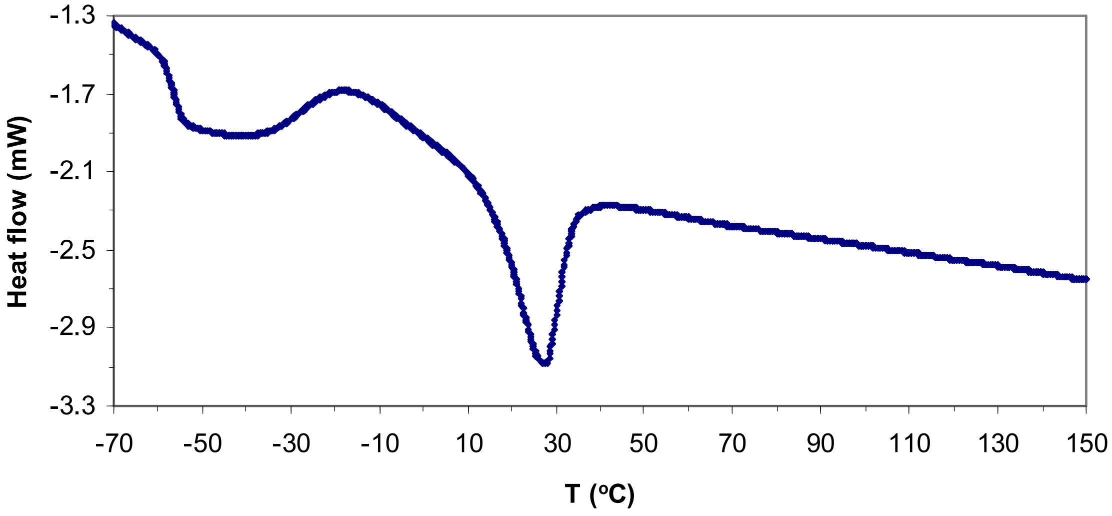


**Figure S5:** DSC trace for the SPUR-2 sample ($T_g$ = -58 °C, and $T_m$ = 27 °C)

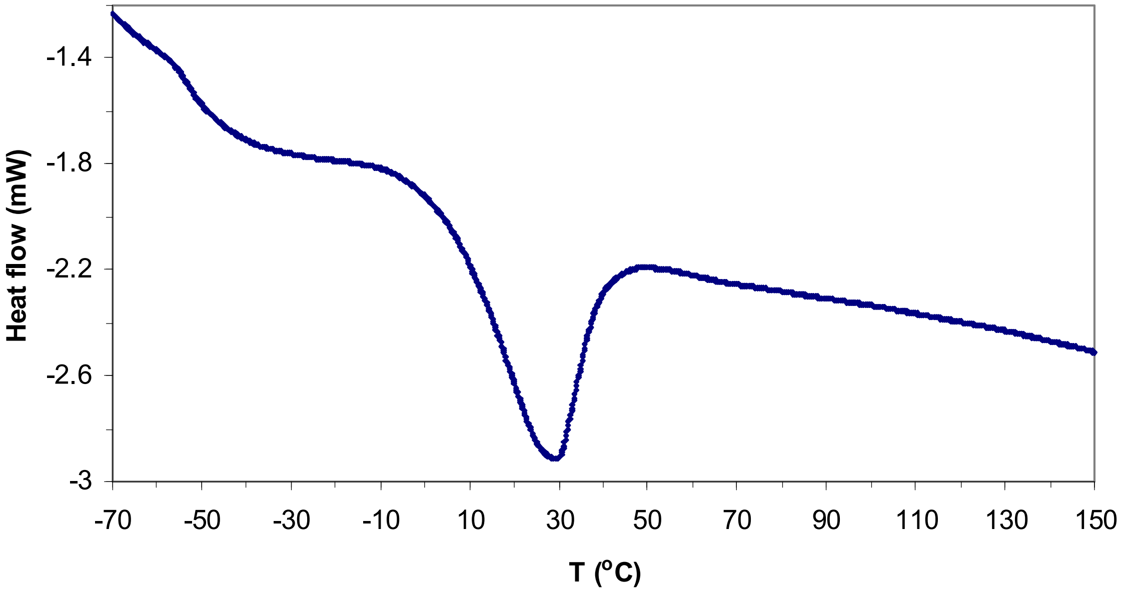


**Figure S6:** DSC trace for the SPUR-4 sample ($T_g$ = -52 °C, and $T_m$ = 30 °C)

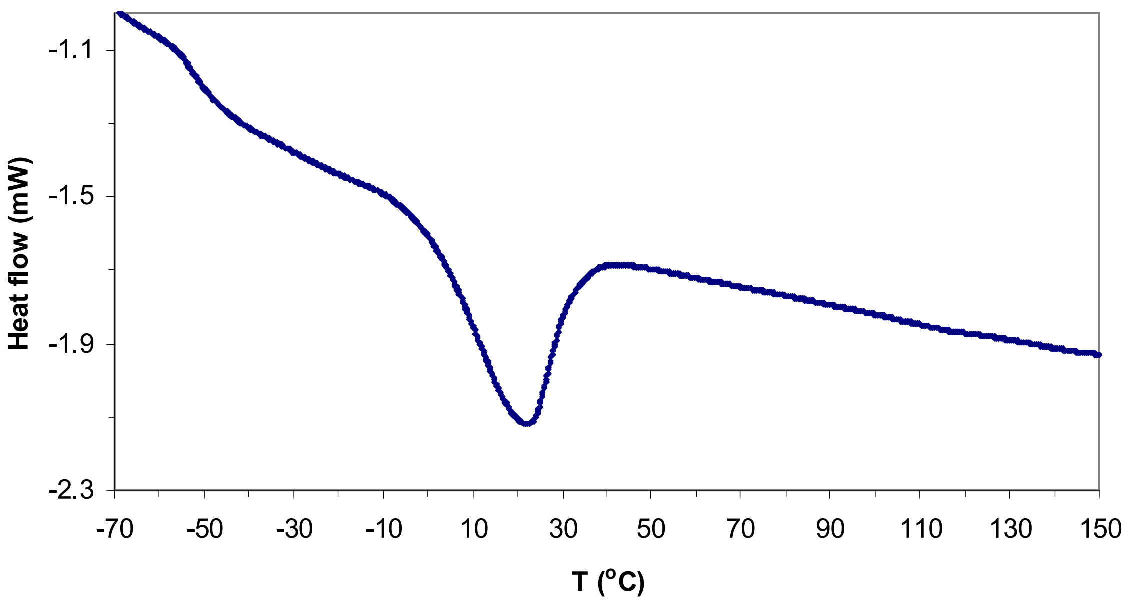


**Figure S7:** DSC trace for the SPUR-5 sample ($T_g$ = -51 $^oC$, and $T_m$ = 23 $^oC$)

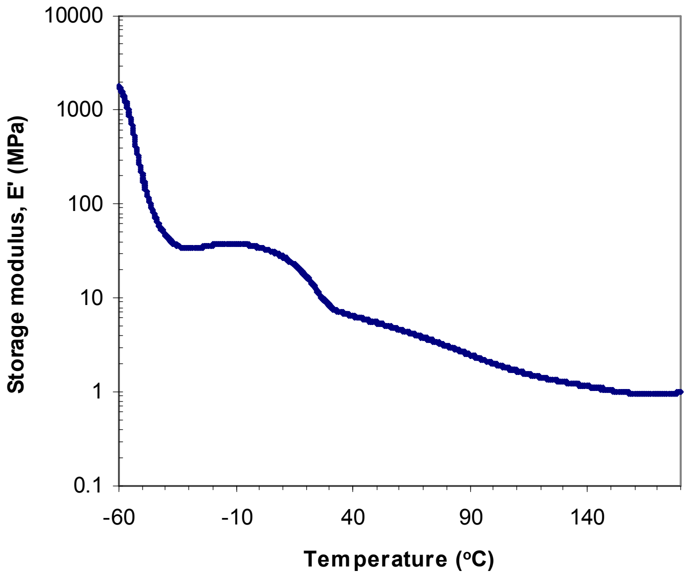


**Figure S8:** DMA trace (variation of the storage modulus (E`) with the temperature in the range of -60 - +180 $^oC$) for the SPUR-1 sample

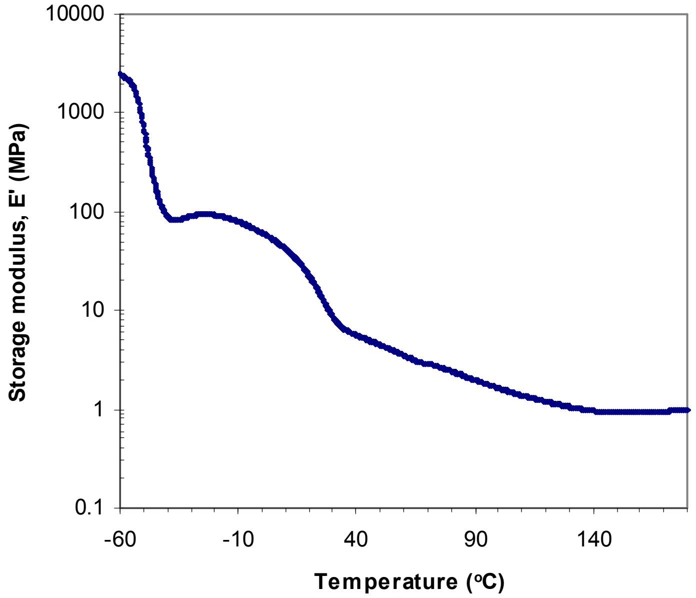


**Figure S9:** DMA trace (variation of the storage modulus (E`) with the temperature in the range of -60 - +180 °C) for the SPUR-2 sample
